# Supplementary material for: Universal structures for adaptation in biochemical reaction networks
Source: Nat Commun. 2023 Apr 20;14:2251. doi: 10.1038/s41467-023-38011-9 (PMC10119132; doi:10.1038/s41467-023-38011-9)
Supplement: Supplementary file 1 — Supplementary Information [file 41467_2023_38011_MOESM1_ESM.pdf]

Supplementary Information for "Universal structures for adaptation in biochemical reaction networks"

Robyn P. Araujo[1*] and Lance A. Liotta[2]

[1]School of Mathematical Sciences, Queensland University of Technology, Australia.
[2]Center for Applied Proteomics and Molecular Medicine, George Mason University, USA.

*To whom correspondence should be addressed: r.araujo@qut.edu.au

Supplementary Text

In this Supplement, we provide full details and analysis of the key technical results summarised in the main paper, along with proofs for all supporting theorems, a range of additional illustrative examples, as well as code in the open-source software *Singular* [5] that can be adapted to any collection of chemical reactions.

The overarching goal of this work is to provide a comprehensive and universal description of all possible Chemical Reaction Network (CRN) structures that can implement the keystone biological function known as Robust Perfect Adaptation (RPA), and in so doing, to propose a definitive algebraic condition that captures the presence of the RPA property in any CRN that possesses it. We also recognise here that all forms of RPA, including the special case known as Absolute Concentration Robustness (ACR), must implement some form of integral control. Our universal characterisation of RPA at the level of intermolecular interactions thereby provides an algorithmic method not only for detecting the RPA property, but also for identifying the hidden

integral controllers, as well as the 'setpoints' of any RPA-exhibiting molecules.

As we show in the pages to follow, providing such an all-encompassing description of RPA at the microscale-level of biological networks - that is, in CRNs, which account for the mathematical graph structure of intermolecular interactions - has required us to reconcile a wide range of disparate mathematical viewpoints, ranging from Chemical Reaction Network Theory (CRNT), control theory and the internal model principle, and algebraic geometry. The essential structure of our mathematical development is as follows:

1. In Section S1 we identify a universal 'kinetic pairing' principle, which captures the essence of RPA in all CRNs that possess the property, and which is codified by our Two-Variable Kinetic Pairing Theorem (Theorem 1, Section S1.4). This theorem guarantees that, for all RPA capable CRNs, the geometric projection of the CRN's steady-state locus onto a state space defined by just *two variables* is captured by a distinguished algebraic invariant that we call an *RPA polynomial*. In this context the term 'variable' has a particular meaning, that we define carefully in Section S1.4.

   In this Section we also introduce readers to the fundamentally important notion of *algebraically independent subnetworks* - a simple yet powerful concept introduced by Martin Feinberg (see Appendix A.6 in [7] for a detailed overview), whose implications for the decomposition of CRNs into independent subnetworks, and ultimately *topological modules*, that are compatible with RPA are recognised for the first time in the present work. This key concept provides a vital ingredient which contributes to a definitive characterisation of RPA-capable CRNs of arbitrarily high deficiency, far beyond the simple deficiency-one ACR-capable CRNs covered by the Shinar-Feinberg theorem [16].

2. In Section S2 we briefly discuss the topological implications of Theorem 1 and, for later reference, make a preliminary connection with previous work on the topological struc-

tures that are known to hold, at the network macroscale, for all RPA-capable networks [1]. Importantly, the universal topological solution to the RPA problem [1] was developed considering only a single external input or disturbance, and did not account for the possibility of *no external inputs*, with alterations in total abundances of the interacting elements (as specified by the initial conditions of the system) constituting the only possible perturbation to the system. More importantly, *and in stark contrast to the present work*, the universal macroscale solution to the RPA problem [1] gives no concrete information as to the CRN structures (at the network microscale) that could engender RPA or satisfy its strict topological requirements.

3. Since most RPA-capable CRNs identified in prior studies identify an RPA polynomial (as we call it here) from only linear coordinate changes (see, for example, Cappelletti et al. [3]), in Section S3 we consider the algebraic properties of CRNs that allow the RPA polynomial (as expressed in Theorem 1) to reside in the rowspan of the CRNs chemical reaction rates (i.e. given by an $\mathbb{R}$-linear combination of the rate equations). For this purpose, we analyse two carefully chosen CRN examples in detail, and demonstrate the existence of several subsidiary polynomial invariants *in the rowspan* for each case, and note the relationship of these polynomial invariants to the overarching topology of the system. We demonstrate via deficiency-preserving transformations of the respective CRN structures that these subsidiary polynomial invariants *combine* to yield the all-important RPA polynomial *within the rowspan* exactly when the invariants are *stoichiometrically dependent*. If the invariants are *stoichiometrically independent*, on the other hand, a *concatenating monomial* is required to reconcile the invariants, thereby introducing a necessary non-linearity to the transformation that produces the RPA polynomial. The detailed analysis of these simple examples provides the reader with an accessible overview of the general principles that characterise RPA-capable CRN structures before we develop these princi-

ples more rigorously and in complete generality in the remaining Sections.

4. In Section S4 we demonstrate, through an analysis of deficiency-increasing reactions within independent CRN subsets, that the mathematical transformation required to extract the all-important RPA polynomial from the CRN rate equations is always *almost linear*, in the sense that the RPA polynomial can always be decomposed into a collection of 'complex linear invariants'. From a control theory viewpoint, these observations lead to the previously unrecognised conclusion that the defining algebraic condition for any RPA-capable CRN can always be decomposed into a collection of *linear* integral controllers, in addition to reconciling the processing of biochemical information at the network microscale (i.e. via the intricate intermolecular interactions that comprise a CRN) with the overarching topological features at the network macroscale [1].

5. In Section S5, we provide the reader with extensively annotated code in the open-source software *Singular* (www.singular.uni-kl.de) [5] to test the RPA capacity of a range of illustrative examples. This code can be adapted readily to the analysis of any CRN. If RPA does obtain for a particular CRN under investigation, an important consequence of the special almost-linear structure of the transformation required to identify the RPA polynomial is that the computational demands of this algorithm do not differ substantially from those of Gaussian elimination (a polynomial-time algorithm).

S1 Mathematical requirements for RPA in general CRNs

Definition of RPA

As we state in the main paper, we consider Robust Perfect Adaptation (RPA) here from the most general possible viewpoint. In particular, a CRN exhibits RPA in the concentration, x, of some molecule exactly when x maintains a constant steady-state value, c (the molecule's

‘setpoint’), *for all steady-states of the system.* The setpoint, c, is a function of some subset of CRN parameters. Moreover, the CRN exhibits RPA (in x) in response to any perturbation or disturbance that does not feature in the functional form of the setpoint, c.

With this very broad definition of RPA in mind, we recognise that there are many possible types of perturbations/disturbances that could alter the steady-state of the system:

1. One or more ‘external’ inputs. A disturbance of this type could arise in the form of an input molecule, I, whose concentration is given by a step function generated by an ‘exosystem’ ($dI/dt = 0$, not included among the CRN reaction rates).

   Alterations in total expression levels (abundances) of the interacting molecules. If this is the only type of perturbation possible, then the CRN is considered a ‘conservative network’, corresponding to a ‘closed’ system (see [7], Chapter 4). In this scenario, the CRN captures the interconversion of molecules among a variety of possible forms. The dynamical system is thereby constrained to evolve within a particular stoichiometric compatibility class (an affine space parallel to the CRN’s stoichiometric subspace – see Section S1.2) determined by the initial conditions. The orthogonal complement to the stoichiometric subspace determines the conservation relations for the CRN, which specify the constant expression levels of the various interacting molecules. Where a molecule can exhibit a fixed concentration across all steady-states when the CRN is subject to perturbations to these total expression levels, this type of RPA is referred to as Absolute Concentration Robustness (ACR, see Chapter 9 in [7]).

2. A disturbance that is encoded in a CRN parameter such as a production rate, μ, of some molecule, M, as reflected in a CRN reaction of the form $\emptyset \xrightarrow{\mu} M$. Note that CRN parameters determined by the intrinsic chemical properties of the interacting molecules (e.g. association/dissociation constants, catalytic constants, etc.) are very stable and not

readily perturbed, except via mutation of the interacting molecules.

In the pursuit of greatest generality, we make no assumptions *a priori* as to which type of network perturbation might affect a CRN, nor do we impose any restrictions on which (or how many) parameters determine the setpoint. Of course, if the setpoint for a putative RPA-capable molecule involves a parameter μ that can be perturbed (see case 3. above) then it is clear that the molecule cannot exhibit RPA in response to that particular disturbance.

S1.1 Notation and mathematical preliminaries

We consider a collection of chemical reactions, involving n interacting molecules, $X_1, \ldots, X_n$, with corresponding concentrations $x_1, \ldots, x_n$. Each reaction is of the form

$$a_1X_1 + \ldots + a_nX_n \to \hat{a}_1X_1 + \ldots + \hat{a}_nX_n, \tag{1}$$

where $a_i, \hat{a}_i \in \mathbb{Z}_{\geq 0}$ are the stoichiometric coefficients of the reactants and products, respectively. In the typical case, $\sum_{i=1}^{n} a_i \leq 2$ for the *reactants* of any reaction, since individual molecules will typically collide pair-wise, sometimes gradually forming large multimolecular complexes prior to a catalytic event or post-translational modification; but in principle, more reactants could contribute to a single reaction, and we make no assumptions *a priori* as to the stoichiometry of reactions.

A standard approach in Chemical Reaction Network Theory (CRNT, see [7] for a detailed primer) is to consider a chemical reaction network (CRN) to consist of three finite sets, $\mathcal{S}$, $\mathcal{C}$, and $\mathcal{R}$, where $\mathcal{S} = \{X_1, \ldots, X_n\}$ is the set of n interacting molecules, or *species*, of the CRN; $\mathcal{C} = \{y_1, \ldots, y_m\}$ is the set of m distinct *complexes* of the CRN, each of which is a multiset on the species, $\mathcal{S}$; and $\mathcal{R} \subset \mathcal{C} \times \mathcal{C}$ is the set of r distinct reactions of the CRN, which define a directed graph on the complexes, each reaction having the form $y_i \to y_j$.

For analytical purposes, it is convenient to introduce an (arbitrary) order on the species

concentrations, setting $\mathbf{x} = [x_1, \ldots, x_n]^T$, which suggests a natural vector representation for each complex $y_k = [a_1, \ldots, a_n]^T \in \mathcal{C}$, where $a_i \in \mathbb{Z}_{\geq 0}$ is the stoichiometric coefficient of the species X_i in the complex y_k. In this way, a dynamical system $f(\mathbf{x}) = \frac{d\mathbf{x}}{dt}$ can be induced by the CRN, by associating a nonnegative real-valued rate function, $\mathcal{K}_{y_i \to y_j}(\mathbf{x})$ to each reaction $y_i \to y_j \in \mathcal{R}$. In particular, a *mass action* dynamical system is induced when $\mathcal{K}_{y_i \to y_j}(\mathbf{x}) = k_{y_i \to y_j} \mathbf{x}^{y_i}$, where $k_{y_i \to y_j} \in \mathbb{R}_{>0}$ is a strictly positive *rate constant* associated to the reaction $y_i \to y_j$, and $\mathbf{x}^{y_i} = x_1^{a_1} x_2^{a_2} \ldots x_n^{a_n}$ for *reactant* complex $y_i = [a_1, \ldots, a_n]^T$.

A CRN thereby induces a dynamical system of the form

$$f(\mathbf{x}) = \frac{d\mathbf{x}}{dt} = \sum_{\mathcal{R}} \mathcal{K}_{y_i \to y_j}(\mathbf{x})(y_j - y_i), \tag{2}$$

which, under the mass action assumption, becomes a polynomial dynamical system of the form

$$f(\mathbf{x}) = \frac{d\mathbf{x}}{dt} = \sum_{\mathcal{R}} k_{y_i \to y_j} \mathbf{x}^{y_i}(y_j - y_i). \tag{3}$$

For mass-action CRNs, it is standard practice to display the rate constant $k_{y_i \to y_j}$ above the corresponding reaction arrow in the chemical reaction representation (given by (1)).

Equation (2) allows for arbitrary kinetics in a CRN, and requires only C^1 smoothness on the component functions $\{f_1, \ldots, f_n\}$. We observe that $\{f_1, \ldots, f_n\} \subset C^1(\mathbb{R}^n)$, the ring of C^1 functions in n real variables. In practice mass-action kinetics, as represented by Equation (3), provides a very general and flexible framework to represent the reaction rates of all possible molecular species in a CRN, including all transient or intermediate multimolecular complexes, and all chemical moieties and molecular activation states. We also note that all key theorems of CRNT, including the deficiency-zero and deficiency-one theorems [7], along with the Shinar-Feinberg theorem for ACR [16], have appealed to the assumption of mass-action kinetics. Mass-action CRNs have also historically served as a framework for deriving approximations (eg. under various quasi-steady-state assumptions (QSSA)), such as the Michaelis-Menten equation

and the Hill equation, to reduce the mathematical complexity of CRNs for analytical purposes. Thus, for the sake of concreteness, and with minimal loss of generality, we focus hereafter on mass-action CRNs, for which $\{f_1, \ldots, f_n\} \subset \mathbb{R}[x_1, \ldots, x_n]$, the ring of polynomials in n variables with real coefficients. In this case, the steady-states of the system constitute a real algebraic variety.

S1.2 Matrix Decomposition of the CRN Mass-Action Equations

For later use, we also recall a useful matrix representation of the mass equations of a CRN, first proposed by Horn and Jackson [11], which extricates the contribution of the CRN's graph structure (considered as a collection of vertices (complexes) and directed edges (reactions)), from the stoichiometry of the reactants and products. As we shall see in Section S4.1, this decomposition is a particularly productive starting point for many important mathematical results in CRNT (see [7]) that are relevant to ACR and RPA.

We consider an arbitrary CRN, $\{\mathcal{S}, \mathcal{C}, \mathcal{R}\}$, comprising n species, m complexes, and r reactions. As noted earlier in Equation (3), the mass action equations of a CRN take the form

$$f(\mathbf{x}) = \frac{d\mathbf{x}}{dt} = \sum_{\mathcal{R}} k_{y_i \to y_j} \mathbf{x}^{y_i}(y_j - y_i), \tag{4}$$

which can be expressed in the equivalent matrix form

$$\frac{d\mathbf{x}}{dt} = Y.\mathcal{L}(G).\psi(\mathbf{x}). \tag{5}$$

Here $Y : \mathbb{R}^m \to \mathbb{R}^n$ is a linear map that associates to each complex $y \in \mathcal{C}$ its corresponding stoichiometry, ie. its multiset of species, whose matrix columns are thus the vector representations of the m CRN complexes: $Y = [y_1, \ldots, y_m]$.

The map $\mathcal{L}(G) : \mathbb{R}^m \to \mathbb{R}^m$ is the Laplacian of the *graph* of the CRN, with complexes $y \in \mathcal{C}$ taken as vertices, and reactions $y_i \to y_j \in \mathcal{R}$ taken as directed edges. $\mathcal{L}(G)$ thus assumes the

matrix form

$$\mathcal{L}(G)_{i,j} = \begin{cases} k_{y_j \to y_i} & \text{if } i \neq j \\ \\ -\sum_{k=1}^{m} k_{y_j \to y_k} & \text{if } i = j. \end{cases}$$

Whereas the entries of Y contain only integers, the entries of $\mathcal{L}(G)$, contain the rate constants of the CRN (and sums of rate constants on its diagonals). The column vector, $\psi(\mathbf{x})$, lists the monomials $\mathbf{x}^{y_i}$ associated to the complexes of the CRN under the mass-action assumption, in an order consistent with the action of the matrix product $Y\mathcal{L}(G)$.

As a minor technicality, Equation (5) includes all complexes of the CRN, including complexes that do not act as a reactant complex for any reaction in the CRN (ie. complexes that represent products only). In this case each column of $\mathcal{L}(G)$ corresponding to such a product-only monomial in $\psi(\mathbf{x})$ contains only zero entries. If convenient, Equation (5) could also be given with these product-only complexes removed from $\psi(\mathbf{x})$, and the corresponding all-zero columns of $\mathcal{L}(G)$ also removed. With slight abuse of notation, noting that the latter version of $\mathcal{L}(G)$ is no longer technically the Laplacian of the CRN graph, either convention may be adopted wherever the explicit consideration of graph structure is not required, since both representations produce the same set of polynomial reaction equations for the network (which contain no product-only monomials).

The matrix decomposition given by Equation (5) explicitly uncouples the graph structure, G, of the CRN (as encoded by $\mathcal{L}(G)$) from the reaction stoichiometry at its vertices (as encoded by Y). In the language of CRNT [7, 16], the connected components of G are referred to as the *linkage classes* of the CRN. A strongly connected component (SCC) of G, being a maximal strongly-connected subgraph of G (see [12]), are referred to as *strong-linkage classes* in CRNT. Moreover, a *terminal strong-linkage class* (or *terminal* SCC) is one in which no complex reacts to a complex in a *different* SCC. Complexes belonging to terminal SCCs are referred to as *terminal complexes*; all other complexes are *non-terminal complexes*. This terminology is

important to the interpretation of the Shinar-Feinberg theroem [16] - a seminal result on ACR.

Finally, we note a key invariant of any CRN known as the *deficiency*, δ, of the CRN. As we shall see in the theorems we present in Section S4.1, the network deficiency encodes important yet subtle information on the (linear) independence of the chemical reactions in relation to the graph structure of the CRN, and will ultimately give us valuable information on polynomials existing in the *rowspan* of the CRN's reaction equations (cf. the much larger space of polynomials in the *ideal* generated by the rate equations - see Section S1.5). In particular, the deficiency of a CRN is increased by one each time a reaction (or linear combination of reactions, involving a particular set of complexes) can be replicated *elsewhere* in the CRN, via a *different* linear combination of reactions, involving a *different* set of complexes. The reactions of a deficiency-zero CRN are thus as linearly independent as they can possibly be given their distribution into linkage classes (see Feinberg [7], Chapter 6). This being the case, each increase in deficiency by one corresponds to the presence of either a 'parallel' pathway in the flow of biochemical information (i.e. an additional feedforward segment) in the CRN, or a feedback loop. As we shall see, this frequently overlooked relationship between CRN deficiency and the overarching topology of the network will allow us to make crucial connections between the fundamental structure of RPA-capable CRNs and the network design principles that are known to exist at the network macroscale for RPA-capable networks [1].

As shown by Feinberg [7], the deficiency of a CRN is easily calculated by the simple formula

$$\delta = m - l - s, \tag{6}$$

where m is the number of complexes in the CRN, l is the number of connected components (linkage classes) of the CRN graph, and s is the dimension of the stoichiometric subspace, S, of the CRN. The stoichiometric subspace is the span of all reaction vectors in the CRN. Thus, s is numerically equal to the number of linearly-independent reaction vectors in the CRN (see

[16] and [7]), and is referred to as the *rank* of the CRN.

S1.3 Algebraically independent subnetworks of a CRN

Since the central aim of this study is to establish a definitive and universal characterisation of all possible RPA-capable CRNs, we will appeal to the notion of 'algebraically independent subnetworks' – a fundamental concept in CRNT that allows CRN reactions to be partitioned into subsets, corresponding to subnetworks, whose steady-states may be determined independently from the rest of the CRN. In the context of RPA, this simple but powerful notion will allow us to distinguish CRN reactions that contribute to the CRN's RPA capacity from reactions that play no role in the CRN's RPA capacity.

The concept of algebraically independent CRN subnetworks was first exploited by Martin Feinberg (see [7]). We provide a brief overview of the essential mathematical ideas here, for later use (see Section S4.2); interested readers can find a more detailed exposition of the mathematical concepts underlying this approach in Appendix 6.A in [7].

Consider a partition of the reaction set, $\mathcal{R}$, of a CRN into p subsets: $\mathcal{R}_1, \mathcal{R}_2, \ldots, \mathcal{R}_p$. This corresponds to a decomposition of the parent CRN $\{\mathcal{S}, \mathcal{C}, \mathcal{R}\}$ into the subnetworks $\{\mathcal{S}, \mathcal{C}_\theta, \mathcal{R}_\theta\}$, $\theta = 1, 2, \ldots, p$, where $\mathcal{C}_\theta = \{y \in \mathcal{C}$: there exists $y \to y' \in \mathcal{R}_\theta$ or $y' \to y \in \mathcal{R}_\theta\}$. In this way, each such subnetwork $\{\mathcal{S}, \mathcal{C}_\theta, \mathcal{R}_\theta\}$ will be characterised by its own stoichiometric subspace, $S_\theta \subset S$, and will have its own rank, $s_\theta =$dim(S_θ), and its own deficiency, δ_θ.

Now,

$$S = S_1 \oplus S_2 \oplus \ldots \oplus S_p \tag{7}$$

exactly when

$$s = s_1 + s_2 + \ldots + s_p. \tag{8}$$

When condition (8) is satisfied, we say that the CRN $\{\mathcal{S}, \mathcal{C}, \mathcal{R}\}$ has been partitioned into p *independent subnetworks* $\{\mathcal{S}, \mathcal{C}_\theta, \mathcal{R}_\theta\}$, $\theta = 1, 2, \ldots, p$.

Now, given a partition of a CRN into independent subnetworks, consider the reaction kinetics of such a CRN (e.g. via the mass action assumption, as given by Equation (4)). Each kinetic subsystem is now characterised by its own species-formation-rate, $f_\theta(x) : \mathbb{R}^n \rightarrow S_\theta$, and for every system state $c \in \mathbb{R}^n$, we have

$$f(c) = f_1(c) + f_2(c) + \ldots + f_p(c). \tag{9}$$

Suppose that c^* is a steady-state of the CRN, in which case $f(c^*) = 0$, and

$$0 = f_1(c^*) + f_2(c^*) + \ldots + f_p(c^*). \tag{10}$$

But given that the p subnetworks are algebraically independent, and therefore satisfy condition (8), we are assured that the direct sum (7) holds. Therefore,

$$f_\theta(c^*) = 0 \tag{11}$$

for every $\theta \in \{1, \ldots, p\}$. As a consequence of (11), if c^* is a steady-state of the parent CRN, it must be a steady-state of each of the independent subnetworks separately.

In closing, we briefly highlight for later reference a crucial connection between the decomposition of a CRN into algebraically independent subnetworks and the overarching topological structure of the CRN. In particular, a partition of a CRN reaction set $\{\mathcal{R}_1, \mathcal{R}_2, \ldots, \mathcal{R}_p\}$ corresponds to a decomposition into independent subnetworks if all reactions of each subset $\mathcal{R}_\theta \in \{\mathcal{R}_1, \mathcal{R}_2, \ldots, \mathcal{R}_p\}$ contain at least one *species* that is unique to that subset - that is, the species is *only* contained in the reactions of that subset, and is absent from the reactions of all other subsets in the partition. That this condition has consequences for the overarching topological structure of the associated CRN can be illustrated through the analysis of the following simple CRN example.

Consider the five-species CRN studied by Cappelletti et al. [3], depicted in Figure S1,

which we consider as a running example throughout this Supplement. The full ('parent') CRN consists of eight reactions, only four of which are linearly independent: C-A, B-C, 2E-B and E-D. The parent CRN thus has a rank of four. In addition, there are ten complexes and four linkage classes, yielding a deficiency of two. One of these deficiency 'units' derives from the fact that the reaction C-A (corresponding to rate constant k_1) is replicated in a different linkage class, with different complexes, via the reaction A-C (corresponding to rate constant k_7). The second of the deficiency 'units' comes from the fact that the reaction E-D (corresponding to rate constant k_8) is replicated in a different linkage class, via different complexes, in the form of the reaction 2D - 2E (corresponding to rate constant k_6). The reactions can be partitioned into two independent subsets, corresponding to Independent Subnetworks 1 and 2 as shown in Figure S1, noting that A and C *only* appear in Independent Subnetwork 1, while D and E *only* appear in Independent Subnetwork 2. Each independent subnetwork has a rank of two, which add to give the rank of the full network ($s = 4$). Independent Subnetwork 1 can be further decomposed into two independent subnetworks, $1(a)$ and $1(b)$, noting that $1(a)$ now contains one independent reaction, A-C, while $1(b)$ contains one independent reaction, B-C. In this decomposition, A is only contained in the reactions of $1(a)$, while B is only contained in the reactions of $1(b)$, and the two reaction subsets correspond to independent subnetworks of Independent Subnetwork 1. Likewise, Independent Subnetwork 2 can be further decomposed into two independent subnetworks, $2(a)$ and $2(b)$, where D only appears in the reactions of $2(a)$, and B only appears in the reactions of $2(b)$, thereby guaranteeing the independence of $2(a)$ from $2(b)$.

We will return to this example in Sections S3.1 and S4.2, and see that the RPA capacity of this CRN is due entirely to the deficiency-one Independent Subnetwork 1, and is completely unaffected by Independent Subnetwork 2. From a topological standpoint, the deficiency of one in Independent Subnetwork 1 corresponds to the presence of two feedforward components,

and thus establishes the essential structure of a Balancer module (see [1]). Moreover, the two subnetworks $1(a)$ and $1(b)$, with deficiency one and zero respectively, each contribute a 'linear complex invariant' [12], which constitute the subsidiary polynomial invariants (see Section S4.2) that together construct the *RPA polynomial* of the CRN (see Section S1.5). Note that only species B is common to both Independent Subnetworks, and thus 'connects' the independent CRN subnetworks. Species B thus plays the role of a 'diverter' [1], and the reactions involving D and E (regulated by B) in Independent Subnetwork 2 constitute 'extramodular' reactions 'upstream' of the Balancer module.

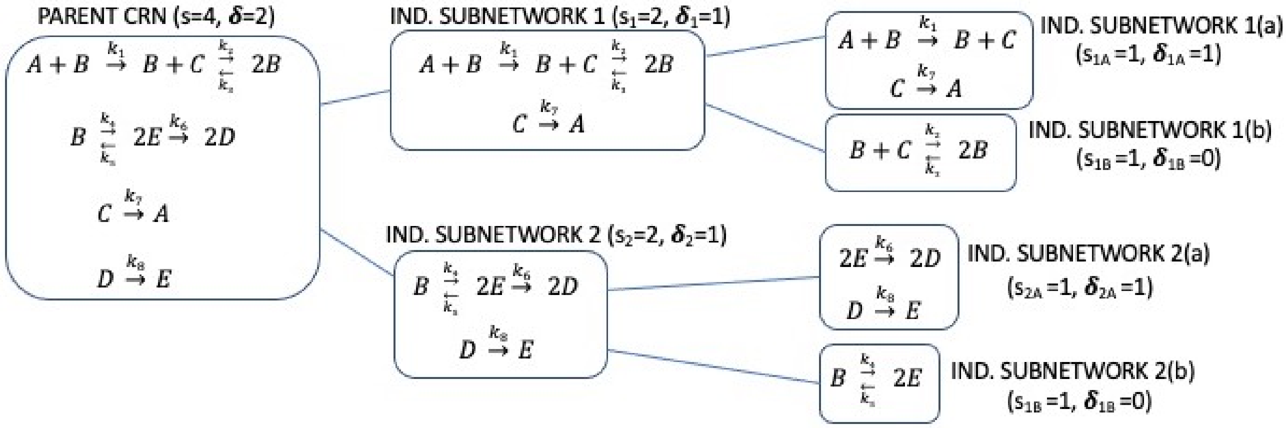

Supplementary Figure S1: Decomposition of the deficiency-two (rank-four) CRN studied by Cappelletti et al. [3] into independent subnetworks. The reactions of the parent network can be partitioned into two subsets, each corresponding to an algebraically-independent subnetwork (Subnetwork 1 and Subnetwork 2), each with a rank of two, and a deficiency of one. Each of these, in turn, can be further decomposed into algebraically-independent subnetworks as shown.

S1.4 RPA vs ACR, and the requirement for integral control

Historically, absolute concentration robustness (ACR) has been studied along very separate lines from robust perfect adaptation (RPA). Importantly, RPA corresponds to a special case

of a defining problem in classical automatic control – namely, the robust asymptotic tracking of a desired trajectory (the system's setpoint), while rejecting unwanted disturbances. In the 1970s, the landmark studies of Francis and Wonham [10, 9] investigated the necessary controller structures to achieve such robust tracking, and established what is now known as the internal model principle (IMP). By this principle, a control system is able to reject exogenous stimuli or disturbances by incorporating within itself a model of the dynamic structure of the stimulus or disturbance. In the face of persistent (constant) disturbances, the internal model must produce constant signals and is equivalent to the requirement for integral control [18].

The study of ACR, by contrast, has largely been studied from the CRNT viewpoint, with little consideration of its connection to control theory, although it has recently been acknowledged that ACR is equivalent to the control theoretic notion of robustness to disturbances in initial conditions [3]. Moreover, ACR has typically been considered in the context of *mass-conservative* CRNs [16, 20], where all constituent molecules are subject to a set of conservation laws, with no transfer of molecules into, or out of, the system, and no net production or degradation of molecules. Mass-conservative CRNs therefore have no *external stimuli* or *inputs*, and are typically perturbed by altering the total abundances (or concentrations) of the constituent molecules - ie. by altering the initial conditions. By contrast, RPA is typically considered in models that are subjected to some external input stimulus, or disturbance, that is distinct from the model variables; in addition, these models often incorporate synthesis and degradation processes, and may not therefore be mass-conservative.

But RPA and ACR both require at least one molecule, or species, to have a steady-state concentration that is 'fixed' across all steady-states of the system, and is thus independent of any external stimulus/disturbance (in the case of RPA) or on total concentrations of molecules (in the case of ACR). In this way, both RPA and ACR have a common mathematical definition; the two phenomena may be distinguished mathematically by the parameters that govern

the system's setpoint. For the general case of RPA, the setpoint may be a function of biochemical rate constants, production/degradation rates, and even the total abundance of constituent molecules (if this quantity is held fixed due to a conservation law). For ACR, on the other hand, the setpoint may only be a function of biochemical rate constants (see Equation (3)), not production/degradation rates, and not total abundances of any constituent molecules. Thus, ACR may be considered a special case of RPA. All classes of RPA, including ACR, thus require some form of integral control [3].

S1.5 The Two-Variable Kinetic Pairing Theorem

As suggested by the discussion in the previous section, RPA (and hence ACR) is a deeply geometric concept, in the sense that the steady-state concentration of at least one species remains fixed, so that the algebraic variety associated to the system is entirely parallel to a coordinate axis or (hyper)plane for any choice of system parameters, and for any system disturbance. This being the case, the capacity for RPA may be detected through the geometric *projection* of the system onto a certain subset of the model species. But for which species subsets is such a projection possible, and which geometric projections reveal the capacity for RPA?

As a prelude to proving our first important claim as to the properties of all RPA-capable CRNs (Theorem 1) we first consider what constitutes a *variable* of the CRN.

Definition 1. Consider a mass-action CRN comprising n species with concentrations $x_1, \ldots, x_n$. Each species contributes to a *variable* of the CRN, which can take one of two possible forms:

1. A monomial, or power product, with the special property that *all* constituent factors (species) *only* appear in the CRN's mass-action equations as factors of this power product. We refer to power products with this property as *boundary variables* of a CRN; the presence of this class of variable in a mass-action CRN corresponds to the existence of *boundary states* (see Remark below).

2. Any species that does not contribute to a boundary variable.

Remark. Generally, we can consider the individual species of the CRN to be the *variables* of the model (case (2) above). But the geometric projection of the algebraic variety associated to a mass-action system onto a subset of variables corresponds algebraically to an elimination process. This being the case, if a particular collection of species *only* appear together in a particular monomial (power product), and in no other form, then no single such species can be eliminated without also eliminating all other species in the collection. The monomial itself must therefore be considered as a single *unit* from the point of view of elimination (and thus, geometric projection). Likewise, a non-zero value at steady-state for any one species in a boundary variable does not guarantee that the system steady-state is contained in the strictly-positive orthant; all members of boundary variables requires non-zero steady-state values to constrain the system state to the positive orthant (hence *boundary* variable). We consider a CRN containing a boundary variable in Example 2 in Section S3, but also give a very simple example of this concept below.

Example of a CRN containing a boundary variable

Consider the following simple four-reaction CRN:

$X_2 + X_3 \xrightarrow{c_1} X_1 + X_4,$

$X_1 + X_2 + X_3 \xrightarrow{c_2} X_5,$

$X_4 \xrightarrow{c_3} X_2,$

$X_5 \xrightarrow{c_4} X_3.$

The mass-action equations for this CRN are:

$$\frac{dX_1}{dt} = f_1 = c_1X_2X_3 - c_2X_1X_2X_3,$$
$$\frac{dX_2}{dt} = f_2 = -c_1X_2X_3 - c_2X_1X_2X_3 + c_3X_4,$$
$$\frac{dX_3}{dt} = f_3 = -c_1X_2X_3 - c_2X_1X_2X_3 + c_4X_5,$$
$$\frac{dX_4}{dt} = f_4 = c_1X_2X_3 - c_3X_4,$$
$$\frac{dX_5}{dt} = f_5 = c_2X_1X_2X_3 - c_4X_5.$$

Notice that the species X_2 and X_3 *only* appear in the form of the product X_2X_3; neither X_2 nor X_3 appears in any other form. Therefore X_2X_3 acts as a 'unit' in this CRN, and must be taken as a variable from the point of view of an elimination problem. Thus, this CRN comprises five species, but four variables: X_1, X_2X_3, X_4 and X_5.

We now proceed to the central concepts underpinning Theorem 1. Let us first consider three key ideals in $\mathbb{R}[x_1, \ldots, x_n]$ of special importance to RPA:

1. I_f, the set of all polynomial consequences of the rate equations for the n interacting molecules. I_f thus contains all polynomials that can be 'reached' by the rate equations in the sense that each polynomial $p \in I_f$ can be expressed in the form $p = h_1f_1 + \ldots + h_nf_n$, for some $h_1, \ldots, h_n \in \mathbb{R}[x_1, \ldots, x_n]$.

2. I_p, the set of all polynomials in the ring $\mathbb{R}[x_1, \ldots, x_n]$ that *vanish* at $x_i = c$, for some variable x_i of the CRN, and some $c \in \mathbb{R}_{>0}$.

3. $I_f \cap I_p$, being the ideal which contains all polynomials in the n variables $x_1, \ldots, x_n$ that vanish at $x_i = c$ *and* can be 'reached' by the rate equations $f_1, \ldots, f_n$.

Let us now introduce an additional, fourth, ideal associated with the system $\{f_1, \ldots, f_n\} \subset \mathbb{R}[x_1, \ldots, x_n]$, which will assist in finding generators of $I_f \cap I_p$, by using the generators of I_f:

4. I_e, an elimination ideal, being the set of all polynomial consequences of $f_1, \dots, f_n$ that contain *only* the variables x_i and x_j, where x_i is the variable being tested for its RPA capacity, and where x_j is some variable that does *not* have the capacity for RPA.

We will now present a definitive test for RPA (necessary and sufficient conditions, on the assumption of system stability) that is valid for *any* collection of chemical reactions, by showing that:

1. I_e is fully contained in $I_f \cap I_p$ (see Supplementary Figure S2).

2. I_e is a principal ideal, with $I_e =< g(x_i, x_j)(x_i - c) >$, where $g(x_i, x_j) \in \mathbb{R}[x_i, x_j]$ is non-vanishing on $(0, X_{itot}) \times (0, X_{jtot})$. X_{ktot} is the maximum possible concentration for the species x_k; in a mass-conservative system X_{ktot} is thus the *total* abundance of all forms of a particular molecule that include x_k as a particular form.

3. The set of polynomials $\{g(x_i, x_j)(x_i - c)$: x_j is a variable that *does not* exhibit RPA$\}$ is a generating set for $I_f \cap I_p$.

Theorem 1 (Two-variable kinetic pairing theorem)**.** Consider a chemical reaction network endowed with polynomial (eg. mass-action) kinetics comprising n species with concentrations $x_1, \dots, x_n$ and corresponding reaction rate functions $\{f_1, \dots, f_n\} \subset \mathbb{R}[x_1, \dots, x_n]$.

The system has the capacity for RPA in the variable x_i if and only if the elimination ideal $<f_1, \dots, f_n> \cap \mathbb{R}[x_i, x_j]$ is a principal ideal generated by a polynomial of the form $g(x_i, x_j)(x_i - c)$, where x_j is any variable of the system that does *not* exhibit RPA, $g(x_i, x_j)$ is a polynomial that is non-vanishing on $(0, X_{itot}) \times (0, X_{jtot})$, and c is a rational function of system parameters (called the RPA 'setpoint').

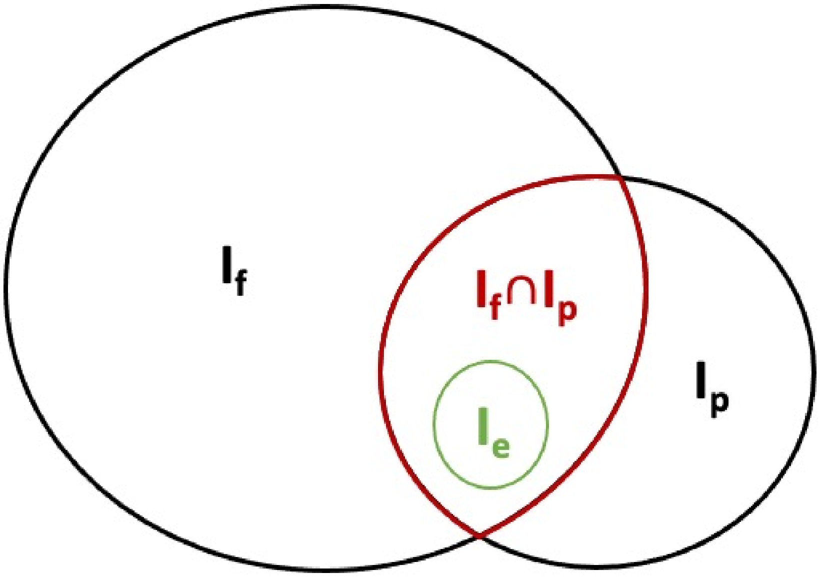

Supplementary Figure S2: An RPA-capable system of polynomial reaction rates is characterised by a special elimination ideal, I_e, that is fully contained in $I_f \cap I_p$.

Proof. A system exhibits RPA in a variable x_i exactly when x_i maintains a constant steady-state value, say c, for all (positive) steady-states of the system. A CRN therefore has the capacity for RPA *only if* there exists some set of polynomials $\{h_1, \ldots, h_n\} \subset \mathbb{R}[x_1, \ldots, x_n]$ such that

$$h_1 f_1 + \ldots + h_n f_n = \hat{p}\,(x_i - c)$$

where $\hat{p} \in \mathbb{R}[x_1, \ldots, x_n]$ is a polynomial that does not vanish in the positive orthant, except possibly at $x_i = c$, and where c is a rational function of the parameters in the rate equations $f_1, \ldots, f_n$.

We first consider the ideal $I_f =< f_1, \ldots, f_n >= \{h_1 f_1 + \ldots + h_n f_n : h_i \in \mathbb{R}[x_1, \ldots, x_n]\}$, and the ideal $I_p =< (x_i - c) >$. If the ideal $I_f \cap I_p$ contains non-zero polynomials, it must have non-zero generators. To establish one such generator, we consider the possible subsets $\bar{x} \subset \{x_1, \ldots, x_n\}$ with the property that $I_f \cap \mathbb{R}[\bar{x}]$ is fully contained in $I_f \cap I_p$. Which subsets $\bar{x}$

can have this property? It is clear that $\bar{x}$ must include x_i, the candidate RPA variable. Suppose $\bar{x}$ contains some other variable x_k, that also exhibits RPA (at $x_k = c'$, say, where c' is a rational function of system parameters, generally distinct from c) - assuming such a variable exists in the CRN in question. In this case, $I_f \cap \mathbb{R}[\bar{x}]$ will contain polynomials in both x_i and x_k that are not contained in $I_f \cap I_p$ - eg. polynomials that vanish at $x_k = c'$. Now suppose instead that $\bar{x}$ contains x_i and more than one variable (say x_j and x_m) that *do not* have the capacity for RPA. Since a perturbation to the CRN that alters the steady state of x_j will also alter the steady-states of other non-RPA capable variables (eg. x_m), $I_f \cap \mathbb{R}[\bar{x}]$ will contain polynomials in x_j and x_m, and that are not contained in $I_f \cap I_p$.

Let us therefore choose $\bar{x}$ to contain *one* non-RPA-capable variable (x_j, say) in addition to x_i. The set $\bar{x}$ now contains two *independent* (uncoupled) variables in the sense that a perturbation to the CRN that alters the steady-state of one of the variables does not affect the steady-state of the other. It follows that $I_f \cap \mathbb{R}[x_i, x_j]$ contains only polynomials that are of the form $p.g(x_i, x_j)(x_i - c)$, where $p \in \mathbb{R}[x_i, x_j]$, and $g \in \mathbb{R}[x_i, x_j]$ is non-vanishing on $(0, X_{itot}) \times (0, X_{jtot})$, other than possibly at $x_i = c$. That is, $I_f \cap \mathbb{R}[x_i, x_j]$ is a principal ideal generated by a polynomial of the form

$$\rho = g(x_i, x_j)(x_i - c).$$

Since this argument holds for *any* variable x_j that does not have the capacity for RPA, it follows that $I_f \cap I_p$ is generated by the set of polynomials

$$\{g(x_i, x_m)(x_i - c) : x_m \text{ a non-RPA-capable variable}\}.$$

Thus a CRN has the capacity for RPA at the variable x_i only if $I_f \cap \mathbb{R}[x_i, x_j]$ is a principal ideal generated by $g(x_i, x_j)(x_i - c)$, for $g(x_i, x_j) \neq 0$ on $(0, X_{itot}) \times (0, X_{jtot})$, except possibly at $x_i = c$, for *any* variable x_j that does not have the capacity for RPA.

The converse is straightforward. If, for any variable x_j that does not have the capacity for RPA, $< f_1, \ldots, f_n > \cap \mathbb{R}[x_i, x_j]$ is generated by a single polynomial of the form $g(x_i, x_j)(x_i - c)$, it follows that $g(x_i, x_j)(x_i - c)$ vanishes at the system's steady-state. If $g(x_i, x_j) \neq 0$ for all possible steady-states of the system, other than possibly $x_i = c$, it follows that $x_i = c$ for all possible steady-states. Hence the CRN has the capacity for RPA at x_i, with setpoint c.

□

Definition 2 (RPA polynomial). A polynomial in two variables, x, y, of the form

$$\rho = g(x, y)(x - c),$$

with $c \in \mathbb{R}$ and $g(x, y) \neq 0$, is called an **RPA polynomial**. Moreover, a CRN is said to *contain an RPA polynomial* exactly when an $(n-2)^{th}$ elimination ideal of the CRN's mass-action equations can be generated by an RPA polynomial (in which case the CRN has the capacity for RPA in the variable x).

Definition 3 (RPA-capacity vs RPA-exhibiting). A CRN has the capacity for RPA in the variable x, with setpoint $x = c$, if it contains an RPA polynomial, $\rho = g(x, y)(x - c)$. If, in addition, $x = c$ is a *stable* steady-state of the CRN's mass-action equations, the CRN also *exhibits* RPA in the variable x.

Remark. 1. Our proof to Theorem 1 appeals to the fact that if we project the polynomial consequences of the system (I_f) onto three variables, two of which (x_j, x_m) do not exhibit RPA, then there will be a generator for the associated elimination ideal containing only x_j and x_m (and not x_i – the RPA-capable variable). Note that a polynomial in x_j and x_m only is *ipso facto* not in I_p (since all polynomials in I_p contain x_i by supposition). The reason for the existence of such a generator for the three-variable elimination ideal is that a system whose steady-states are determined by a number of *independent* (constant)

disturbances, or input stimuli, must be able to adapt to each disturbance individually, corresponding to a single degree of freedom. Once the value of any one non-RPA variable (x_j, say) is specified, as a consequence of setting the magnitudes of the various possible disturbances/inputs, then the value of any other non-RPA variable (e.g. x_m) is thereby also specified. The value of x_i, by contrast, is not determined through an alteration to *any* such single degree of freedom since it exhibits RPA, and is independent of these disturbances.

2. The fact that the key elimination ideal in Theorem 1 is generated by a polynomial in a specific number of variables, namely *two*, is key. Previous consideration of the concept of *constrained integral control* [3, 20] has posited the notion of a *constrained integrator* [3] of the form $r(t)(x - c)$, where x is the RPA variable, c its setpoint, and $r(t)$ is *some* function of system variables. But identifying that we must project the system onto exactly two variables immediately converts the question of RPA capacity to a well-defined geometric projection problem, and hence an algebraic elimination problem, for *any* CRN.

3. If the setpoint, c, depends only on biochemical rate constants and not on total concentrations (X_{ktot}) or production/degradation rates of any of the molecules, the system also has the capacity for ACR in the variable x_i.

   Moreover, although we note in the statement and proof of Theorem 1 that c is a rational function of biochemical parameters, in the special case of an RPA variable that is a *boundary variable*, the setpoint of the corresponding RPA *species* may be an algebraic, rather than a rational, function of parameters. Consider, for instance, the one-species system: $\frac{dx}{dt} = c_1 - 2c_2x^2$; here x appears only in the form of the monomial x^2, which is therefore a boundary variable. The 'setpoint' for the boundary variable, x^2, is thus $\frac{c_1}{2c_2}$ - a rational function of parameters - while the setpoint for the species x is $\sqrt{\frac{c_1}{2c_2}}$.

4. Although it may not be clear *a priori* which variables to select for these two special choices, x_i and x_j, in an algorithmic test for RPA capacity, we will see in the next section that Theorem 1 has important topological implications. Thus, decomposition of the CRN into algebraically independent subnetworks, alongside a consideration of the topology of each subnetwork (via an analysis of its deficiency - see Sections S1.2 and S1.3) can readily suggest a suitable choice for the candidate RPA-capable variable x_i, as well as a judicious choice for the non-RPA-capable variable, x_j.

5. With a well-defined algebraic elimination problem now at hand, with two candidate variables selected for the geometric projection, the existence of an RPA polynomial as the sole generator of the relevant elimination ideal can always be tested *in principle* via the computation of a suitable Gröbner basis [4]. Unfortunately, the problem of computing Gröbner bases (eg. via Buchberger's algorithm) *for* **general** *polynomial systems* is well known to be NP-Hard [15, 14, 19], so without any special simplifying structure to the collection of polynomials, we can have no assurance that any Gröbner basis-computing algorithm will terminate on a practical time-frame. However, we will show in the Sections to follow that RPA-capable CRNs **do indeed** have a special structure that allows the relevant Gröbner basis to be computed rapidly, on a time-scale comparable to Gaussian elimination. This is because the fundamental 'building blocks' of the RPA polynomial (i.e. its *subsidiary polynomial invariants*), *always* reside in the *rowspan* of the CRN reaction equations (see Sections S3 and S4), and the RPA polynomial can always be constructed from these by multiplying each by a 'concatenating monomial' (where necessary, depending on the stoichiometric dependence of the invariants, see Section S3), and adding the resulting polynomials. As a consequence, the most computationally-demanding component of the resulting algorithm for an RPA-capable CRN (cf. general polynomial systems) is the computation of these subsidiary polynomial invariants (also known as 'complex

linear invariants' [12]) from the CRN reaction equations. Note, in particular, that where only $\mathbb{R}$-linear combinations of the rows of $Y\mathcal{L}(G)$ are computed, the S-polynomials of Buchberger's algorithm are identical to the row combinations obtained during Gaussian elimination (applied to the matrix $Y\mathcal{L}(G)$). Gaussian elimination is well-known to be a polynomial-time algorithm, which terminates rapidly even for relatively large numbers of variables (or complexes, in the case of CRNs).

We note that the ACR-capacity of the deficiency-two EnvZ-OmpR motif was demonstrated via computation of a Gröbner basis, in an ad-hoc manner, by Perez Millan et al. [13]. But without knowing which, or how many, variables *generally* characterise the RPA-encoding elimination ideal, and without establishing the special 'almost linear' structure of the mathematical transformation required to identify the RPA polynomial *in any RPA-capable CRN*, this approach does not generalise, and cannot be applied to the systematic analysis of large and complicated CRNs (e.g. in the context of metabolism [6]) for which candidate RPA-capable molecules have not already been posited from experimental evidence.

6. The statement of Theorem 1 also encompasses special cases where $g(x_i, x_j)$ is zero order in x_i, or x_j, or both. With judicious choice of x_i and x_j, as we explore in the next section, we have most commonly obtained a polynomial $g(x_i, x_j)$ that is zero order in x_i, for a wide range of CRNs (some of which are included in the present paper as illustrative examples). Less commonly, we have also found examples of CRNs where $g(x_i, x_j)$ is zero order in *both* x_i *and* x_j (as is the case for the antithetic integral control motif [2] - see Section S1.6).

Before proceeding to an examination of the topological implications of Theorem 1 (Section S2), as well as the decomposition of RPA polynomials into a collection of 'complex linear

invariants' (obtained through linear coordinate changes (Sections S3 and S4)), and the consequences of this special 'almost linear structure' of RPA-capable CRNs for the implementation of integral control (Section S4), we first illustrate the application of Theorem 1 to the network depicted in Supplementary Figure S3 (also considered in Figure 1 in the main paper).

S1.6 Example: RPA in a Two-Node Opposing Set Involving Antithetic Integral Control

In Supplementary Figure S3 we present a collection of chemical reactions, which incorporates a sub-collection of reactions (noted in red) that constitute a motif known as *antithetic integral control*. With only the red reactions considered, the law of mass action would induce two rate equations, $\dot{x_1} = k_1 R - k_2 x_1 x_2$ and $\dot{x_2} = k_3 - k_2 x_1 x_2$, such that $\dot{x_1} - \dot{x_2} = k_1(R - k_3/k_1)$, which is an RPA polynomial with $g(x_1, x_2) = k_1$ (which is zero-order in both x_1 and x_2 in this particularly simple case) and setpoint $c = k_3/k_1$. But with the additional (black) reactions included, it is not immediately clear if RPA will still obtain (at R) for this more extensive collection of reactions. By computing the Gröbner basis (see Singular code provided in Section S5), and selecting $x_i = R$ and $x_j = x_2$ for the two-variable projection, we obtain the RPA polynomial

$$\begin{aligned}\rho_1 &= k_1^2 k_6 k_7 x_2 R^2 - (2k_1 k_3 k_6 k_7 + k_1 k_4 k_6 k_8) x_2 R + (k_3^2 k_6 k_7 + k_3 k_4 k_6 k_8) x_2 \\ &\quad - k_1 k_4 k_5 k_7 R + (k_3 k_4 k_5 k_7 + k_4^2 k_5 k_8) \\ &= \left(R - \left[\frac{k_3}{k_1} + \frac{k_4 k_8}{k_1 k_7}\right]\right)\left(k_1^2 k_6 k_7 x_2 \left[R - \frac{k_3}{k_1}\right] - k_1 k_4 k_5 k_7\right),\end{aligned}$$

demonstrating that the CRN exhibits RPA at R with setpoint $c = \frac{k_3}{k_1} + \frac{k_4 k_8}{k_1 k_7}$.

To illustrate that we can project onto *any* non-RPA-capable molecule to test for RPA, not

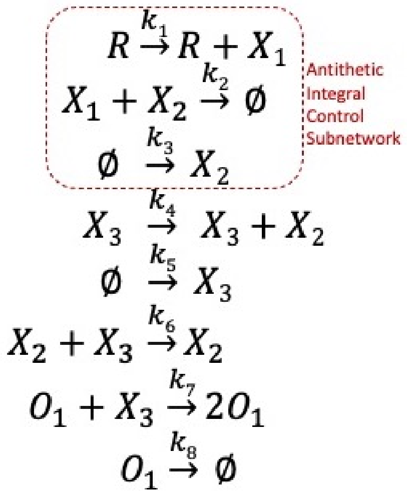

Supplementary Figure S3: Chemical reaction network incorporating antithetic integral control as a sub-motif.

just x_2, we repeat the test with $x_j = x_1$ which yields

$$\begin{aligned}\rho_2 &= k_1k_2k_4k_5k_7x_1R - (k_2k_3k_4k_5k_7 + k_2k_4^2k_5k_8)x_1 - k_1^3k_6k_7R^3 + (2k_1^2k_3k_6k_7 + k_1^2k_4k_6k_8)R^2 \\ &\quad - (k_1k_3^2k_6k_7 + k_1k_3k_4k_6k_8)R \\ &= k_1k_7\left(R - \left[\frac{k_3}{k_1} + \frac{k_4k_8}{k_1k_7}\right]\right)\left(k_2k_4k_5x_1 - k_1^2k_6R\left[R - \frac{k_3}{k_1}\right]\right),\end{aligned}$$

and with $x_j = O_1$, which yields

$$\begin{aligned}\rho_3 &= k_1k_7O_1R - (k_3k_7 + k_4k_8)O_1 \\ &= k_1k_7\left(R - \left[\frac{k_3}{k_1} + \frac{k_4k_8}{k_1k_7}\right]\right).\end{aligned}$$

With the choice of $x_j = x_3$ (and $x_i = R$), we find that an RPA polynomial no longer obtains, demonstrating that this CRN has the capacity for RPA at x_3 as well as R. Indeed, repeating the

test with $x_i = x_3$, and $x_j \in \{x_2, x_1, O_1\}$, we obtain, respectively,

$$\begin{aligned}\rho_4 &= k_6k_7x_2x_3^2 - k_6k_8x_2x_3 - k_5k_7x_3 + k_5k_8 \\ &= k_7\left(k_6x_2x_3 - k_5\right)\left(x_3 - \frac{k_8}{k_7}\right),\end{aligned}$$

$$\begin{aligned}\rho_5 &= k_2k_5k_7x_1x_3 - k_2k_5k_8x_1 - k_4k_6k_7x_3^3 + \left(k_4k_6k_8 - k_3k_6k_7\right)x_3^2 + k_3k_6k_8x_3 \\ &= k_7\left(x_3 - \frac{k_8}{k_7}\right)\left(k_2k_5x_1 - k_6x_3(k_4x_3 + k_3)\right),\end{aligned}$$

and

$$\begin{aligned}\rho_6 &= k_7O_1x_3 - k_8O_1 \\ &= k_7O_1\left(x_3 - \frac{k_8}{k_7}\right).\end{aligned}$$

Each of these three RPA polynomials demonstrates that the setpoint for x_3 is $\frac{k_8}{k_7}$. Thus, for RPA variable R, the ideal $I_f \cap I_p =< \rho_1, \rho_2, \rho_3 >$. For RPA variable x_3, $I_f \cap I_p =< \rho_4, \rho_5, \rho_6 >$.

S2 The RPA Polynomial and the Topological Hierarchy of Invariants

In the previous section we proved that all CRNs that are capable of exhibiting RPA at a particular variable (x_i) are characterised by an elimination ideal in *exactly two* variables (x_i and x_j) that can be generated by a single polynomial, called an RPA polynomial, with the particular form $\rho = g(x_i, x_j)(x_i - c)$. Moreover, we proved that if a CRN has the capacity for RPA at variable x_i, a corresponding RPA polynomial exists for *every* variable x_j that does *not* have the capacity for RPA.

We now consider the consequences of Theorem 1 for the underlying network structure, or topology, of RPA-capable CRNs, along with the critical question of making suitable choices for

x_i and x_j in an algorithmic test for RPA-capacty.

First, since $\rho = 0$ for any network steady-state, the existence of an RPA polynomial in a CRN's mass-action equations implies that the activating (or *upregulating*), influence of the non-RPA-capable variable (x_j) is exactly matched, or *paired*, with its inhibitory (or *downregulating*), influence on the RPA-variable, x_i, via the function g. We refer to this feature of the RPA polynomial, and the regulation of the underlying CRN, as *kinetic pairing*. With this observation in mind, we note that there are two network (ie. topological) interpretations of ρ: (i) x_j is a regulator of x_i; or (ii) x_j is regulated by x_i. See Supplementary Figure S4.

From this, the topological significance of the RPA polynomial is clear: If x_j is a *regulator* of x_i, and its upregulating and downregulating influences on the regulated variable x_i are exactly *paired* as described above, this implies an overarching network structure that corresponds to a Balancer module (see Supplementary Figure S4B, where x_j plays the role of a *diverter variable*, and x_i (the RPA variable) is a *connector variable*). See [1] for a comprehensive explanation of Balancer modules. Likewise, if x_j is *regulated* by x_i, and the upregulating and downregulating influences on the regulating (RPA) variable x_i are exactly *paired* as described above, this implies an overarching network structure that corresponds to a Opposer module (see Supplementary Figure S4A, where x_j plays the role of an *opposer variable*, and x_i (the RPA variable) is the unique regulator of the Opposer module). See [1] for a comprehensive explanation of Opposer modules. Indeed, we note the striking similarity between an RPA polynomial, ρ, and both *connector kinetics* (which are required in 'Balancer Modules' [1]) and *opposer kinetics* (which are required in 'Opposer Modules') as presented in our previous general solution to the RPA problem at the network macroscale (see [1]).

While, in practice, some trial and error may be necessary in the selection of elimination variables for highly complex CRNs, these topological observations suggest natural choices for x_i and x_j. In particular, if the CRN is topologically of Balancer-type, any molecule with the

function of a diverter variable is a judicious choice for x_j, the non-RPA-capable variable, while a molecule regulated by x_j via a number of parallel pathways (ie. a connector molecule) should be assigned to x_i as the candidate RPA-capable variable. By contrast, if the CRN is topologically of Opposer type, a molecule acting as the sole regulator of the collection of opposer molecules is a suitable choice for x_i, while any member of a collection of opposer molecules makes a suitable choice for x_j.

These principles will be clarified through the analysis of a range of additional examples in the remainder of this Supplement. In particular, we will show that the elimination ideal associated to a CRN's RPA polynomial has a very special structure, which reveals a universal implementation of *integral control* for all RPA-capable CRNs.

S3 RPA Polynomials in the Rowspan of a CRN

Given that all RPA-capable CRNs have a two-variable RPA-polynomial, ρ, in the ideal associated to the system's rate equations - ie. $\rho = h_1 f_1 + \ldots + h_n f_n$, for some $\{h_1, \ldots, h_n\} \subset \mathbb{R}[x_1, \ldots, x_n]$ - we now wish to clarify the algebraic, and ultimately topological, significance of a set of h_i's that can perform the elimination task required for the system to 'reach' ρ. We first consider a preliminary question: Under what circumstances are $h_i \in \mathbb{R}$? That is, when does an RPA-capable CRN possess an RPA polynomial in the rowspan of the system, where ρ is an $\mathbb{R}$-linear combination of the polynomial rate equations?

To address this question, we first examine two different RPA-capable CRNs whose RPA polynomial is *not* in the rowspan of the system. In each case, we consider a simple deficiency-preserving modification to the network to allow the same RPA polynomial to exist in the system's rowspan. These examples are sufficiently simple for the elimination process to be executed manually, but are sufficiently involved to point to deep and general properties of the elimination problem that exists in all RPA-capable CRNs.

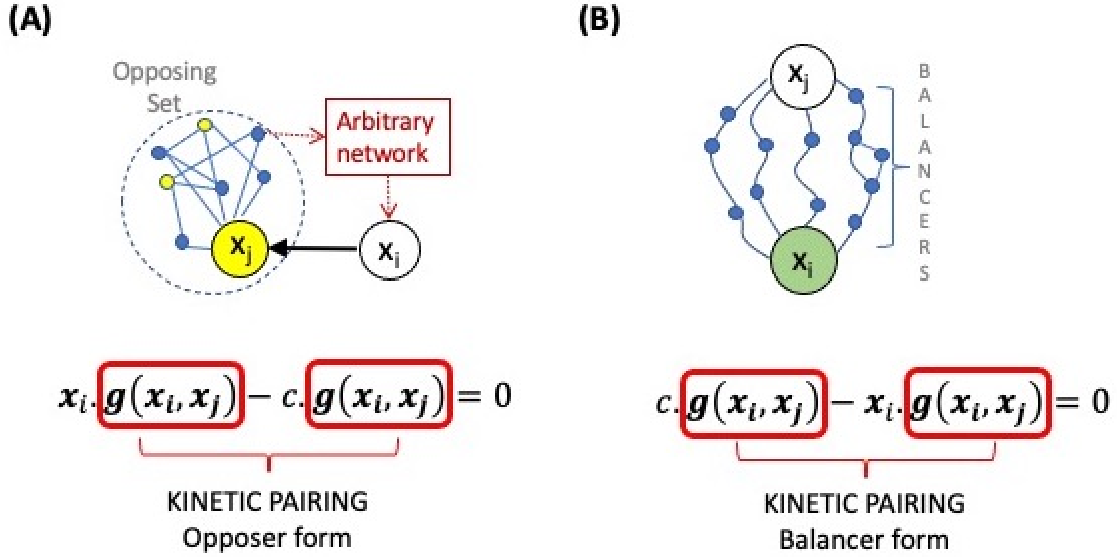

Supplementary Figure S4: (Presented as Figure 4 in the main paper). The principle of **kinetic pairing**, via a *pairing function* $g(x_i, x_j)$. (a) Kinetic pairing in an Opposer module: Here the *regulator* molecule, x_i, has the capacity for RPA, while the influence of the *regulated* molecule, x_j, which does not exhibit RPA, is balanced (paired) in both its upregulatory and downregulatory contributions. (b) Kinetic pairing in a Balancer module: In this case, the *regulated* molecule, x_i, has the capacity for RPA, while the influence of the *regulating* molecule, x_j, which does not exhibit RPA, is paired in both its upregulatory and downregulatory contributions. Full details on Opposer and Balancer modules, at the level of the network macroscale, are given in [1].

S3.1 Example 1

Cappelletti et al. [3] discuss an ACR-capable toy model comprising biochemical interactions among five molecules, A, B, C, D and E, for which it was shown that no $\mathbb{R}$-linear combination of the system's rate equations can take the form $r.(A^\gamma - q)$, for some polynomial $r \in \mathbb{R}[A, B, C, D, E]$ and some $\gamma, q \in \mathbb{Q}_{>0}$, even though the system could be shown to exhibit ACR in the molecule A.

We depict this toy model in Supplementary Figure S5, and distinguish two independent subnetworks (see Section S1.3) via different colours - black for one, and red for the other. Note

that each of these two independent subnetworks has a deficiency of one. In the first independent subnetwork, the deficiency of one arises from the two parallel segments for the interconversion between A and C (see Supplementary Figure S1). Indeed, we observe from the set of chemical reactions that the species B inhibits A by direct binding and conversion to C (reaction at rate k_1). In addition, B regulates the production of C (reactions at rates k_2 and k_3), while C in turn produces A (reaction at rate k_7). Thus B both upregulates A (via C) and downregulates A. For this reason, the collection of chemical reactions is topologically a Balancer module [1]; in particular, B, the mediator of the two parallel feedforward contributions, and the molecule common to both independent subnetworks, acts as the 'diverter' of the Balancer module (see [1]). A is the 'connector' species, and thus has the potential to exhibit ACR (RPA). We depict this topological relationship schematically in Supplementary Figure S5 (center). We further note that in the second independent subnetwork, the deficiency of one arises from the feedback loop consisting of two separate reactions, involving distinct sets of complexes, controlling the interconversion between E and D. Indeed, B regulates E, which regulates D, which in turn regulates E through a separate reaction (feedback component), as indicated in Supplementary Figure S5. From the decomposition into algebraically independent subnetworks, it is clear that the feedback interactions involving D and E do not play any role in the RPA capacity of the system. Intriguingly, this conclusion corresponds exactly to what is expected from a strictly topological viewpoint [1], since network interactions upstream of a diverter node play no role in the RPA-conferring properties of a Balancer module [1], but are still entirely compatible with the topology of a Balancer Module.

In Supplementary Figure S5 (right) we present a modified version of the Cappelletti model [3] - one which preserves the decomposition into two independent deficiency-one subnetworks, and hence the topological structure of the CRN, but is constructed from a slightly different collection of chemical reactions.

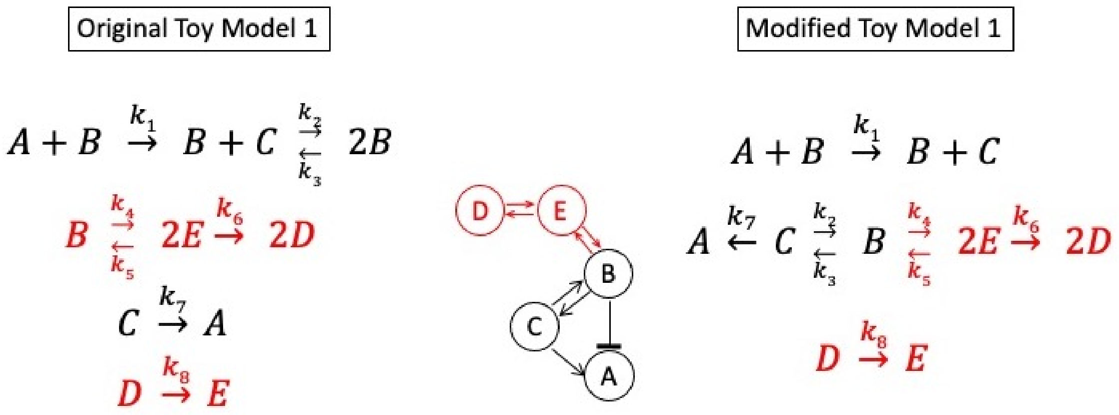

Supplementary Figure S5: A toy CRN proposed by [3] (left) along with a modified version (right). Both CRNs have the underlying topology of a Balancer Module, and thus have the potential for RPA capacity at A. Both CRNs have a deficiency of two, and thus elude the Shinar-Feinberg theorem [16].

Under the law of mass action, both the original and modified models have identical reaction rates for species A, D, and E, namely

$$\dot{A} = k_7 C - k_1 AB, \tag{12}$$

$$\dot{D} = 2k_6 E^2 - k_8 D, \tag{13}$$

$$\dot{E} = k_8 D - 2(k_5 + k_6)E^2 + 2k_4 B. \tag{14}$$

For the **original model** (Supplementary Figure S5 (left)), the reaction rates for B and C are

$$\dot{B} = k_2 BC - k_3 B^2 + k_5 E^2 - k_4 B, \tag{15}$$

$$\dot{C} = k_1 AB - k_7 C - k_2 BC + k_3 B^2, \tag{16}$$

while for the **modified model** (Supplementary Figure S5 (right)), the reaction rates for B and

C are, instead,

$$\dot{B} = k_2C - k_3B + k_5E^2 - k_4B, \tag{17}$$

$$\dot{C} = k_1AB - k_7C + k_3B - k_2C. \tag{18}$$

Since both CRN models are topologically of balancer type, with A as the connector species [1], they have the *potential* to be RPA-capable at species A. Whether or not they *do*, in fact, have the capacity for RPA depends on the intricate molecular details of the CRN, leading to the presence or absence of an RPA polynomial in the ideal generated by the system equations. Since B is topologically a diverter species [1], it is natural to project the system onto the two species A and B, to look for an RPA polynomial of the form

$$\rho = g(B)(A - \kappa). \tag{19}$$

The balancing nature of both models, whereby B regulates A directly, and indirectly via C, is captured by the equation for $\dot{A}$ (Equation (12)), from which it is clear that if C is proportional to B at steady-state, then the system will contain an RPA polynomial in its steady-state ideal.

That both models are indeed characterised by a steady-state concentration of C in direct proportion to that of B is revealed by the polynomial ($\dot{A} + \dot{C}$). Indeed, for the modified model,

$$\dot{A} + \dot{C} = k_3B - k_2C. \tag{20}$$

From this it is clear that an RPA polynomial exists *in the rowspan* of the modified model, since the variable to be eliminated (C) exists as a linear monomial in both (12) and (20), which allows these two equations to be combined through linear combination to yield the needed RPA

polynomial, ie.

$$k_2\dot{A} + k_7(\dot{A} + \dot{C}) = k_2k_7C - k_1k_2AB + k_7k_3B - k_2k_7C \tag{21}$$

$$= -k_1k_2B\left(A - \frac{k_7k_3}{k_1k_2}\right), \tag{22}$$

indicating that, for $B^* \neq 0$, $A^* = (k_7k_3)/(k_1k_2)$. (Here and elsewhere in this Supplement, we indicate steady-states by a superposed asterisk.) In this way, we 'pass' the invariant given by (20) to the invariant given by (12) through the operation of linear combination. The RPA polynomial and its two component invariants are linearly dependent. In fact, if we simply remove the 'unnecessary' (from an RPA viewpoint) reactions from this CRN (indicated in red in Supplementary Figure S5 (right), the remaining CRN has deficiency one, and can be shown to exhibit ACR (at A) by the Shinar-Feinberg theorem.

For the original model, on the other hand,

$$\dot{A} + \dot{C} = -k_2BC + k_3B^2 = -k_2B\left(C - \frac{k_3}{k_2}B\right), \tag{23}$$

so $C^* = (k_3/k_2)B^*$ - a relationship of direct proportion - so that an RPA polynomial of the form (19) can exist in the ideal of the system. But in this case, no *linear* combination of $\dot{A}$ and $(\dot{A}+\dot{C})$ can eliminate C to construct the needed RPA polynomial. This is because in Equation (23), C is contained only in the monomial BC, whereas in Equation (12), C exists only as a linear monomial. Reconciling the two invariants, then, to construct the RPA polynomial requires a 'concatenating monomial': Equation (12) must first be multiplied by the linear monomial B to produce

$$B\dot{A} = -k_1AB^2 + k_7BC. \tag{24}$$

Equation (24) clearly resides in the ideal generated by the CRN's reaction equations, but not in the rowspan, and now incorporates C by the monomial BC as in the invariant (23).

Now (23) and (24) can be combined through linear combination to produce the requisite

RPA polynomial,

$$
\begin{aligned}
k_2 B\dot{A} + k_7(\dot{A} + \dot{C}) &= -k_1 k_2 AB^2 + k_2 k_7 BC - k_2 k_7 BC + k_7 k_3 B^2, \\
&= -k_1 k_2 AB^2 + k_7 k_3 B^2, \\
&= -k_1 k_2 B^2 \left(A - \frac{k_7 k_3}{k_1 k_2} \right),
\end{aligned}
$$

Thus, for the original model, we 'pass' the balancer invariant $(\dot{A}+\dot{C})$ to the connector invariant $\dot{A}$ by first applying a concatenating monomial (B) to $\dot{A}$ in order for the elimination step to be executed.

The key distinction between the two models, from the point of view of the RPA-promoting structure of their reaction graphs, and thus of their rate equations, is one of stoichiometry. For the original model, the balancer polynomial (Equation (23)) incorporates the species C from the reaction $B + C \to 2B$, whereas for the modified model, the balancer polynomial (Equation (20)) incorporates C from the reaction $C \to B$. Thus, since C appears in the connector polynomial (Equation (12)) from the reaction $C \to A$, the original model requires a concatenating monomial (B, applied to the connector polynomial) to reconcile the stoichiometries of the reactions contributing to its balancer and connector polynomials. Thus, we say that the balancer and connector polynomials for the original model are *stoichiometrically independent*. No such concatenating monomial is required for the modified model, since the reactions contributing to its balancer and connector polynomials are already stoichiometrically compatible from an elimination standpoint.

S3.2 Example 2

In the Supplementary Information to [16], Shinar and Feinberg present a CRN of an EnvZ-OmpR motif of deficiency two (see Supplementary Figure S6 (left), and Figure 2b in our main article) which, unlike its deficiency-one counterparts in the main paper of [16], fall outside the

scope of the Shinar-Feinberg theorem. Nevertheless, the model is known to exhibit ACR in the species Y_p.

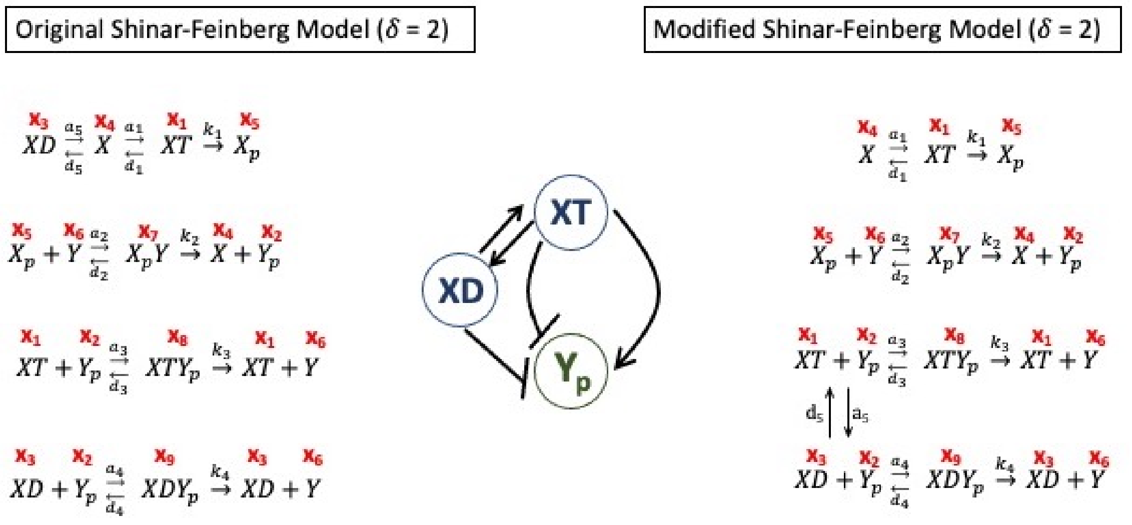

Supplementary Figure S6: A deficiency-two CRN of the EnvZ(X)-OmpR(Y) osmoregulation system studied by Shinar and Feinberg [16] (left). As shown (centre) this CRN is, topologically, a Balancer Module. A modified (hypoethetical) version of the CRN is also presented (right), which preserves both topology and deficiency.

Shinar and Feinberg's deficiency-two model of the EnvZ-OmpR CRN is represented in Supplementary Figure S6 (left), with the species $\{XT, Y_p, XD, X, X_p, Y, X_pY, XTY_p, XDY_p\}$ denoted by $\{x_1, \ldots, x_9\}$, respectively, in order to simplify the rate equations. It is straightforward to show that this particular CRN cannot be decomposed further into algebraically independent subnetworks since the deficiency of two arises from the fact that there are *three* independent collections of reactions, corresponding to three feedforward segments, that can be combined linearly to produce the same reaction: $Y - Y_p$ (the conversion from Y to Y_p). The first of these feedforward segments comes from the first two linkage classes of the CRN, while the second and third feedforward segments are contributed by the third and fourth linkage classes, respectively. Note that each of these reaction collections is mediated by a species within the set of

non-terminal complexes in the first linkage class (XD, X, or XT). For this reason, this particular ACR-capable network is, topologically-speaking, a Balancer Module, with XD, X, or XT as diverter species, as depicted schematically in Supplementary Figure S6 (center).

For the sake of concreteness, we select XT as the diverter species: XT directly downregulates Y_p through direct binding and enzymatic conversion back to Y. XT also indirectly downregulates Y_p via XD (a balancer species) through the binding of XD to Y_p and enzymatic conversion to Y. In addition, X_T upregulates Y_p via X_p (a balancer species), with the binding of X_p to Y catalysing the transfer of a phosphate group to produce Y_p. A similar argument holds when selecting XD or X as a candidate diverter species instead, in which case XT will function as a balancer species. In any case, there are three parallel pathways leading from the diverter species to Y_p (the connector species [1]), conferring the potential for RPA (and in this case ACR, more specifically) to this collection of reactions.

Under the law of mass action, this chemical reaction graph structure induces the following nine rate equations:

$$f_1 = \dot{XT} = \dot{x_1} = a_1x_4 - (d_1 + k_1)x_1 - a_3x_1x_2 + (d_3 + k_3)x_8, \tag{25}$$

$$f_2 = \dot{Y_p} = \dot{x_2} = k_2x_7 - a_3x_1x_2 + d_3x_8 - a_4x_3x_2 + (k_4 + d_4)x_9, \tag{26}$$

$$f_3 = \dot{XD} = \dot{x_3} = d_5x_4 - a_5x_3 - a_4x_3x_2 + (k_4 + d_4)x_9, \tag{27}$$

$$f_4 = \dot{X} = \dot{x_4} = a_5x_3 + d_1x_1 - (a_1 + d_5)x_4 + k_2x_7, \tag{28}$$

$$f_5 = \dot{X_p} = \dot{x_5} = k_1x_1 - a_2x_5x_6 + d_2x_7, \tag{29}$$

$$f_6 = \dot{Y} = \dot{x_6} = d_2x_7 - a_2x_5x_6 + k_3x_8 + k_4x_9, \tag{30}$$

$$f_7 = \dot{X_pY} = \dot{x_7} = a_2x_5x_6 - (d_2 + k_2)x_7, \tag{31}$$

$$f_8 = \dot{XTY_p} = \dot{x_8} = a_3x_1x_2 - (d_3 + k_3)x_8, \tag{32}$$

$$f_9 = \dot{XDY_p} = \dot{x_9} = a_4x_2x_3 - (d_4 + k_4)x_9. \tag{33}$$

Notice that both x_5 and x_6 *only* appear in this system of equations as components of the monomial x_5x_6, which thereby acts as a single 'unit', and is therefore a boundary variable of the model. There are thus eight variables in this model: $x_1, x_2, x_3, x_4, x_5x_6, x_7, x_8, x_9$.

Now consider our **modified version** of Shinar and Feinberg's model in Supplementary Figure S6 (right). Like Shinar and Feinberg's original model (Supplementary Figure S6 (left)), this is topologically a Balancer Module, with XT, XD and X all potential diverter species, and Y_p the connector species. Under the law of mass action, f_2, f_5, f_6, f_7, f_8 and f_9 for the modified system are identical to their counterparts in the original model. By contrast, f_1, f_3 and f_4 are now altered in the modified system, and take the forms

$$f_1 = \dot{XT} = \dot{x_1} = a_1x_4 - (d_1 + k_1)x_1 - (a_3 + d_5)x_1x_2 + (d_3 + k_3)x_8 + a_5x_3x_2, \tag{34}$$

$$f_3 = \dot{XD} = \dot{x_3} = d_5x_1x_2 - (a_5 + a_4)x_2x_3 + (k_4 + d_4)x_9, \tag{35}$$

$$f_4 = \dot{X} = \dot{x_4} = d_1x_1 - a_1x_4 + k_2x_7. \tag{36}$$

An examination of the rate equation f_2 reveals the balancing nature of this motif in both the original and modified forms:

$$f_2 = \underbrace{k_2x_7}_{\substack{\text{upregulation by } XT \\ \text{via } X_p \text{ and } X_pY}} \quad \underbrace{-a_3x_1x_2 + d_3x_8}_{\substack{\text{downregulation by} \\ \text{conversion to } Y \text{ by } XT}} \quad \underbrace{-a_4x_3x_2 + (k_4 + d_4)x_9}_{\substack{\text{downregulation by} \\ \text{conversion to } Y \text{ by } XD}}. \tag{37}$$

We now consider the additional row operations on the system equations that bring Equation (37) closer to the form of an RPA polynomial as follows:

First, for both the original and modified CRNs, the rate equation f_8 indicates that x_8 is directly proportional to the monomial x_1x_2 at steady state. The second grouping of terms in Equation (37) can therefore be absorbed into a single term via the identity

$$\frac{d_3}{d_3 + k_3} f_8 = \frac{a_3d_3}{d_3 + k_3} x_1x_2 - d_3x_8.$$

Likewise, the rate equation f_9 indicates that x_9 is directly proportional to the monomial $x_2 x_3$ at steady state, enabling the third grouping of terms in Equation (37) to be absorbed into a single term via the identity

$$\frac{d_4}{d_4+k_4} f_8 = \frac{a_4 d_4}{d_4+k_4} x_2 x_3 - d_4 x_9.$$

In addition, it is clear that x_1 is proportional to x_7 at steady state, since

$$f_5 + f_7 = k_1 x_1 - k_2 x_7.$$

Therefore, the rowspan of the system contains the polynomial

$$\hat{p} = f_2 + \frac{d_3}{d_3+k_3} f_8 + \frac{d_4}{d_4+k_4} f_9 + f_5 + f_7 = k_1 x_1 - \frac{a_3 k_3}{d_3+k_3} x_1 x_2 - \frac{a_4 k_4}{d_4+k_4} x_2 x_3. \tag{38}$$

The crucial feature of Equation (38) is that if x_1 is proportional to x_3 at steady-state, then $\hat{p}$ will assume the form of an RPA polynomial.

Now, for the modified system, we observe that x_1 and x_3 are indeed proportional at steady-state, provided $x_2 \neq 0$, since

$$f_3 + f_9 = d_5 x_1 x_2 - a_5 x_2 x_3 \tag{39}$$

- a balancer condition [1]. Moreover, Equation (39) can be directly incorporated (by linear combination) into Equation (38) to yield the RPA polynomial

$$\begin{aligned} a_5 \hat{p} - \frac{a_4 k_4}{d_4+k_4} (f_3 + f_9) &= a_5 k_1 x_1 - \frac{a_5 a_3 k_3}{d_3+k_3} x_1 x_2 - \frac{a_4 k_4 d_5}{d_4+k_4} x_1 x_2, \\ &= a_5 k_1 x_1 - \gamma x_1 x_2, \\ &= -\gamma x_1 \left(x_2 - \frac{a_5 k_1}{\gamma} \right), \end{aligned}$$

where $\gamma = \left(\frac{a_5 a_3 k_3}{d_3+k_3} + \frac{d_5 a_4 k_4}{d_4+k_4} \right)$.

For the original system, on the other hand, we note that x_1 and x_3 are proportional at steady

state from the identities

$$f_3 + f_9 = d_5 x_4 - a_5 x_3$$

and

$$f_1 + f_8 = a_1 x_4 - (d_1 + k_1)\, x_1,$$

which gives

$$a_1(f_3 + f_9) - d_5(f_1 + f_8) = d_5(d_1 + k_1)x_1 - (a_1 a_5)x_3. \tag{40}$$

Thus, we now observe a critical difference between the original and modified systems from the point of view of their RPA capacity: both systems are RPA-capable (indeed ACR capable) since they both contain the polynomial $\hat{p}$ in their rowspan, and each system contains a 'balancer polynomial' in its rowspan that constrains x_1 to be proportional to x_3 at steady state, thereby allowing $\hat{p}$ to assume the form of an RPA polynomial. But the two systems differ in how this additional balancer condition is realised *stoichiometrically*. And, as a consequence, the two systems differ in how the two conditions (the connector polynomial, $\hat{p}$, and the respective balancer polynomial) can be combined *algebraically* to produce the RPA polynomial.

Importantly, in both Example 1 and Example 2 (Sections S3.1 and S3.2), the balancer polynomials and connector polynomials required to construct the respective RPA polynomials were in the *rowspan* of the CRN's mass action equations. In the next section, we will see that this is *always* true of RPA-capable CRNs of balancer type: the connector polynomial, and any requisite balancer polynomials, are *always* in the rowspan of the CRN's mass action equations. A similar result obtains for CRNs of opposer type, whose mass-action equations *always* contain opposer polynomial(s) in the rowspan.

S4 RPA Invariants and a Universal Implementation of Integral Control for all RPA-Capable CRNs

In this section, we further explore the significance of a CRN's deficiency in the context of RPA capacity, and show that for *all* RPA-capable CRNs, the rowspan of the system contains a connector polynomial along with all required balancer polynomials (if the CRN is topologically of Balancer-type), or one or more opposer polynomials (if the CRN is topologically of Opposer-type).

The standard control theory viewpoint is to identify a (generally nonlinear) coordinate change that enables the system to be partitioned into two components [18] - one being the 'internal model', and the other being the remainder of the system. In the viewpoint we propose, by contrast, we partition the system into (generally) *more than two* components. In particular, the internal model is distributed over a number of elements (invariants) in our formulation, each of these invariants being obtained by a linear coordinate change (i.e. via a linear combination of the CRN's rate equations). These individual invariants may then be combined nonlinearly through 'monomial concatenation' - that is, through multiplication by a suitable monomial in order to reconcile each such invariant with the 'next' invariant in the CRN's topological structure - to obtain the all-important RPA polynomial (see previous Section).

We note that, although there should always exist some single nonlinear coordinate change that yields the RPA polynomial *in principle*, for any mass action system, identifying such a single coordinate change *in practice* may be extremely difficult in all but the very simplest RPA-capable CRNs. (See, for instance [17] for an example of identifying a single nonlinear coordinate change for a very simple RPA-capable motif). Of greater concern is the fact that, even where a suitable nonlinear coordinate change can be identified, this transformation, in and of itself, cannot reveal a universal picture of the essential structural principles by which RPA is

transacted through CRNs *in general*.

By contrast, the integral control formulation we present here provides a definitive picture of *all possible* RPA-capable CRNs. Moreover, the fact that the transformation required to obtain an RPA polynomial is *almost linear*, in the sense that a limited number of special polynomial invariants - each of which is obtained through *linear* transformation - are combined to yield the RPA polynomial, means that the existence of an RPA polynomial can be quickly checked through direct computation of a suitable Gröbner basis with an elimination monomial ordering. In fact, it is only on account of this special 'almost linear' transformation that the feasibility of this approach is guaranteed: as we noted in our remarks following Theorem 1, the *general* problem of computing Gröbner bases, for *general* collections of polynomials, is known to be NP-Hard. But the fact that the RPA polynomial can always be computed by combining a collection of polynomials *in the rowspan of the system* means that the algorithm that extracts the RPA polynomial from the CRN reaction equations does not differ substantially from Gaussian elimination. Moreover, even for an arbitrary choice of two projection variables, our statement of Theorem 1 offers opportunities for additional computational efficiency when computing a suitable Gröbner basis insofar as only *one* particular elimination ideal is required (i.e. that comprising the two chosen variables). Without prior knowledge of this particular property of the RPA polynomial, a full set of *all* elimination ideals for the system would be required, which necessitates an expensive lexicographic monomial ordering in the execution of the algorithm. With only one elimination ideal required, a more efficient block ordering may be chosen, with a comparatively fast (e.g. degree reverse lexicographic) ordering imposed on all but the two chosen variables. In any case, this simple and direct algorithmic test for RPA capacity also yields the setpoint of the system, along with a candidate set of elimination polynomials $(h_1, \ldots, h_n)$ which reveal how many (nonlinear) concatenation steps are required for the CRN in question.

We now provide a mathematical justification for our claim that any RPA-capable CRN has

connector and balancer polynomials (for Balancer-type CRNs) or opposer polynomials (for Opposer-type CRNs) in the *rowspan* of its mass-action equations. We first briefly review a number of key known theorems, as well as several new theorems, relevant to our claim that linear coordinate changes are always sufficient to identify the key subsidiary invariants of any RPA-capable CRN.

S4.1 Known Results

Proposition 1. For any mass-action CRN, with reaction rates given by $f(\mathbf{x}) = Y.\mathcal{L}(G).\psi(\mathbf{x})$, the following holds

$$\dim(\ker \mathrm{Y}\mathcal{L}(\mathrm{G})) - \dim(\ker \mathcal{L}(\mathrm{G})) \leq \delta.$$

Proof. See Section 6 of [8]. □

Corollary 1. Consider a mass-action CRN with either δ = 0, or $\delta = 1$. Then,

$$\dim(\ker \mathrm{Y}\mathcal{L}(\mathrm{G})) - \dim(\ker \mathcal{L}(\mathrm{G})) = \delta.$$

Proof. See Proof for Lemma S3.20 in Supplementary Information of [16]. □

Remark. In other words, it follows that $\dim(\ker \mathrm{Y}\mathcal{L}(\mathrm{G})) = \dim(\ker \mathcal{L}(\mathrm{G}))$ for deficiency-zero networks, and that $\dim(\ker \mathrm{Y}\mathcal{L}(\mathrm{G})) = \dim(\ker \mathcal{L}(\mathrm{G})) + 1$ for deficiency-one networks.

The next theorem, originally proved in [8], appeals to the standard terminology that the support of a vector $\mathbf{z}$, $\mathrm{supp}(\mathbf{z})$, is the collection of non-zero elements of $\mathbf{z}$.

Proposition 2. Consider a CRN with t terminal SCCs,$\{\Lambda^1, \ldots, \Lambda^t\}$. The kernel of $\mathcal{L}(G)$ contains a basis $\{b^1, \ldots, b^t\}$ with the property that supp(b^i) $= \Lambda^i$, for $i \in \{1, \ldots, t\}$.

Proof. See Appendix of [8]. □

Remark. The dimension of the kernel of $\mathcal{L}(G)$ is numerically equal to the number of terminal SCCs of the CRN. For each terminal SCC, Λ^i, the associated basis vector, b^i, has non-zero entries corresponding to the complexes contained in Λ^i, and zero entries elsewhere. In other words, each basis vector has its support on the complexes of a single terminal SCC of the CRN graph.

Lemma 1. Consider an n-species mass-action CRN of deficiency one with at least one non-terminal complex, and a steady state $\mathbf{c} \in \mathbb{R}^n_{>0}$ in the positive orthant. Then,

$$\mathrm{ker}\mathrm{Y}\mathcal{L}(\mathrm{G}) = \mathrm{ker}\mathcal{L}(\mathrm{G}) \oplus \eta,$$

where $\eta = \{\alpha\psi(\mathbf{c}) : \alpha \in \mathbb{R}\}$.

Proof. This follows straightforwardly from Corollary 1 and Proposition 2 above. In particular, since $\mathbf{c}$ is a steady-state of the system, the set η forms a one-dimensional vector space contained in $\mathrm{ker}\mathrm{Y}\mathcal{L}(\mathrm{G})$ that is linearly independent of $\mathrm{ker}\mathcal{L}(\mathrm{G})$, since all vectors in $\mathrm{ker}\mathcal{L}(\mathrm{G})$ have support in the terminal complexes (only), whereas $\psi(\mathbf{c})$ has support on all complexes (which include at least one non-terminal one, by supposition), since $\mathbf{c}$ is in the positive orthant. □

Theorem 2 (Shinar-Feinberg)**.** Consider a mass-action system of deficiency one that admits a positive steady state. If, in the network, there are two non-terminal complexes that differ only in species S, then the system has absolute concentration robustness (ACR) in S.

Proof. See Supplementary Information for [16] □

S4.2 Polynomials in the Rowspan of a Mass-Action CRN

We now provide an alternative version of Shinar-Feinberg's theorem, drawing on arguments developed by Cappelletti et al. [3] (see Theorem D.1 in [3]), which leads to a key result on an

important class of polynomials (more specifically, *binomials*) that always reside in the rowspan of certain mass-action CRNs.

Theorem 3. Consider a deficiency-one mass-action CRN with state vector $\mathbf{x} \in \mathbb{R}^n$, that admits a steady-state, $\mathbf{c} \in \mathbb{R}^n$, in the positive orthant. For any two non-terminal complexes, with corresponding monomials $\psi_i(\mathbf{x})$ and $\psi_j(\mathbf{x})$, there exists some $\mathbf{a} \in \mathbb{R}^n$ such that

$$\mathbf{a}^T \frac{d\mathbf{x}}{dt} = \alpha_1 \psi_i(\mathbf{x}) - \alpha_2 \psi_j(\mathbf{x}),$$

for some pair of rational functions (of system parameters), α_1 and α_2.

Proof. We consider the vector of monomials $\psi(\mathbf{x})$, whose component $\psi_k(\mathbf{x})$ corresponds to complex k ($k \in \{1, \ldots, m\}$). Now, consider any two distinct non-terminal complexes of the CRN, say y_i and y_j, with corresponding monomials $\psi_i(\mathbf{x})$ and $\psi_j(\mathbf{x})$.

Consider a vector $\rho \in \mathbb{R}^m$ with exactly two non-zero entries, namely $\rho_i = \psi_j(\mathbf{c})$ and $\rho_j = \psi_i(\mathbf{c})$. Recall that for deficiency-one CRNs, $\mathrm{kerY}\mathcal{L}(\mathrm{G}) = \mathrm{ker}\mathcal{L}(\mathrm{G}) \oplus \eta$ (where η is as defined in Lemma 1). It is clear that ρ is orthogonal to all vectors in η, i.e $\rho \in \eta^{\perp}$. Moreover, since any vector $b \in \mathrm{ker}\mathcal{L}(\mathrm{G})$ can be expressed in the form $a_1 b^1 + \ldots + a_t b^t$, where the support of each $b^i \in \{b^1, \ldots, b^t\}$ is on terminal complexes (see Proposition 2), it is clear that $\rho \in (\mathrm{ker}\mathcal{L}(\mathrm{G}))^{\perp}$. Thus, since $\rho \in \eta^{\perp}$ and $\rho \in (\mathrm{ker}\mathcal{L}(\mathrm{G}))^{\perp}$, it follows from Lemma 1 that $\rho \in (\mathrm{kerY}\mathcal{L}(\mathrm{G}))^{\perp}$. Moreover, since ρ lies in the orthogonal complement to the kernel (nullspace) of $Y\mathcal{L}(G)$, it follows that ρ^T is in the rowspan of $Y\mathcal{L}(G)$. Thus, there exists some $\mathbf{a} \in \mathbb{R}^n$ such that $\mathbf{a}^T \frac{d\mathbf{x}}{dt} = \alpha_1 \psi_i(\mathbf{x}) - \alpha_2 \psi_j(\mathbf{x})$, with $\alpha_1 = \rho_i = \psi_j(\mathbf{c})$ and $\alpha_2 = \rho_j = \psi_i(\mathbf{c})$. Moreover, since the matrix entries of $Y\mathcal{L}(G)$ contain linear combinations of the system rate constants, it follows that $\alpha_1 = \rho_i$ and $\alpha_2 = \rho_j$ are rational functions of system parameters. □

Corollary 2. For a deficiency-one mass-action CRN that admits a positive steady-state, whose RPA polynomial is its connector polynomial (if the CRN is topologically of balancer type) or its

(single) opposer polynomial (if the CRN is topologically of opposer type), then the RPA polynomial is guaranteed to be the rowspan of the system, and its RPA capacity may be determined by the Shinar-Feinberg theorem [16].

Theorem 4. Consider a deficiency-zero mass-action CRN with state vector $\mathbf{x} \in \mathbb{R}^n$, that admits a steady-state, $\mathbf{c} \in \mathbb{R}^n$, in the positive orthant. For any two complexes within the *same* **terminal** SCC, with corresponding monomials $\psi_i(\mathbf{x})$ and $\psi_j(\mathbf{x})$, there exists some $\mathbf{a} \in \mathbb{R}^n$ such that

$$\mathbf{a}^T \frac{d\mathbf{x}}{dt} = \alpha_1 \psi_i(\mathbf{x}) - \alpha_2 \psi_j(\mathbf{x}),$$

for some pair of rational functions (of system parameters), α_1 and α_2.

Proof. This result follows straightforwardly using the same reasoning as the proof of Theorem 3. In this case, $\mathrm{ker} \mathrm{Y}\mathcal{L}(\mathrm{G}) = \mathrm{ker}\mathcal{L}(\mathrm{G})$ since $\delta = 0$, and thus, for any steady-state $\mathbf{c}$, $\psi(\mathbf{c}) \in \mathrm{ker}\mathcal{L}(\mathrm{G})$ is expressible in the form $a_1 b^1 + \ldots + a_t b^t$, where the support of each $b^i \in \{b^1, \ldots, b^t\}$ is on terminal complexes (see Proposition 2). We consider two distinct complexes, y_i and y_j, with corresponding monomials $\psi_i(\mathbf{x})$ and $\psi_j(\mathbf{x})$, both from the *same* terminal SCC, Λ^k. Let b^k be the basis vector of $\mathrm{ker}\mathcal{L}(\mathrm{G})$ corresponding to Λ^k.

Now, taking a vector $\rho \in \mathbb{R}^m$ with exactly two non-zero entries, namely $\rho_i = \psi_j(\mathbf{c})$ and $\rho_j = \psi_i(\mathbf{c})$, it is clear that ρ is orthogonal to b^k. And since b^k has disjoint support from all other $t-1$ basis vectors of $\mathrm{ker}\mathcal{L}(\mathrm{G})$ (see Proposition 2), it follows that $\rho \in (\mathrm{ker}\mathcal{L}(\mathrm{G}))^{\perp}$. Thus, $\rho \in (\mathrm{ker}\mathrm{Y}\mathcal{L}(\mathrm{G}))^{\perp}$, and ρ^T is in the rowspan of $Y\mathcal{L}(G)$. Thus, there exists some $\mathbf{a} \in \mathbb{R}^n$ such that $\mathbf{a}^T \frac{d\mathbf{x}}{dt} = \alpha_1 \psi_i(\mathbf{x}) - \alpha_2 \psi_j(\mathbf{x})$, with $\alpha_1 = \rho_i = \psi_j(\mathbf{c})$ and $\alpha_2 = \rho_j = \psi_i(\mathbf{c})$. Moreover, since the matrix entries of $Y\mathcal{L}(G)$ contain linear combinations of the system rate constants, it follows that $\alpha_1 = \rho_i$ and $\alpha_2 = \rho_j$ are rational functions of system parameters. □

Remark. It follows from the arguments developed for Theorems 3 and 4 that for two distinct distinct complexes, y_i and y_j, with corresponding monomials $\psi_i(\mathbf{x})$ and $\psi_j(\mathbf{x})$: (i) if y_i and y_j

are taken from two *different* terminal SCCs, or (ii) if y_i is non-terminal while y_j is taken from some terminal SCC, then there *cannot* be a binomial of the form $\alpha_1\psi_i(\mathbf{x}) - \alpha_2\psi_j(\mathbf{x})$ in the rowspan of $Y\mathcal{L}(G)\psi(\mathbf{x})$. See Cappelletti et al [3], Proposition C.5.

From Theorems 3 and 4, the contents of the rowspan of a CRN's mass-action equations, and the RPA-capacity of the CRN, may easily be determined for deficiency-zero and deficiency-one CRNs. But once $\delta > 1$, no such simple conclusions may be drawn in complete generality since the dimension of the kernel of $Y\mathcal{L}(G)$ now exceeds the dimension of the kernel of $\mathcal{L}(G)$ by *at least* δ (as opposed to *exactly* δ, as was the case when $\delta = 1$ or 0). Nevertheless, it is clear that RPA is possible for CRNs with $\delta > 1$, and there are many known examples, including Examples 1 and 2 examined in the preceding section, and Figures 2 and 3 in the main paper.

To proceed further to the general principles of RPA-capable CRNs of arbitrary deficiency (δ), we recognise that there are two distinct ways for a network to increase its deficiency while preserving the capacity for RPA:

1. Through deficiency-increasing reactions that belong to algebraically independent subset(s) other than the independent subset(s) that actually implement RPA in the CRN. We considered an example of this scenario in Supplementary Figure S1 in Section S1.3, and again in Example 1 in Section S3.1, where the RPA-capacity was due entirely to one of the two independent subsets. Independent subsets of CRN reactions that do not contribute to RPA may therefore be discarded from the analysis, and RPA capacity may be determined from the remaining independent subsets. Note that for CRNs for which multiple independent subnetworks contribute independently to RPA, these independent subnetworks correspond to distinct *modules* from a topological perspective (see [1]).

2. Through deficiency-increasing reactions in an algebraically independent subset of the CRN reactions that cannot be decomposed further into independent subsets. In contrast

to the previous case *ut supra*, the added deficiency-increasing reactions cannot simply be discarded since they are now part of the RPA mechanism itself. In fact, the fact that a higher deficiency ($\delta > 1$) subnetwork cannot be further decomposed necessarily means that the added deficiency (beyond unity) corresponds to additional independent collections of reactions (involving distinct sets of complexes) that replicate the *same* reaction combinations as the first 'unit' of deficiency in that subset, and thus can only correspond topologically to additional (i.e. more than two) parallel pathways within a Balancer module (and/or possibly feedback loops embedded into those pathways). Where higher deficiencies arise from a collection of distinct, yet interconnected, feedback loops that contribute to RPA, these feedback-promoting reactions can be partitioned into independent subsets (since multiple *distinct* feedback loops are involved). This scenario corresponds topologically to an *opposing set* (see Section S4.4).

We now consider each of these possibilities in turn, through the analysis of some illustrative examples.

S4.3 Deficiency-increasing reactions that do not contribute to RPA

Consider, for example, the well-established antithetical integral control mechanism (Supplementary Figure S7), which is topologically of Opposer type:

$$R \xrightarrow{k_1} R + X_1$$

$$X_1 + X_2 \xrightarrow{k_2} \emptyset \xrightarrow{k_3} X_2$$

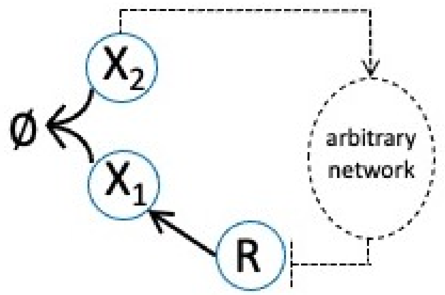

Supplementary Figure S7: Antithetic integral control, as proposed by Briat et al. [2].

This reaction mechanism comprises five complexes ($m = 5$), two linkage classes ($l = 2$) and has a rank of two ($s = 2$), so that the deficiency of this portion of the CRN - excluding any additional chemical reactions that may be added into the overarching feedback loop as indicated in Supplementary Figure S7 - is one. From this it is clear from Theorem 3 that, considering the two non-terminal complexes R and ϕ (with corresponding monomials R and 1, respectively), the rowspan of the mass-action system will contain a polynomial of the form $\alpha_1 R - \alpha_2$ - which is an opposer polynomial as well as the RPA polynomial of the system, which indicates that the CRN has the capacity for RPA at R. Indeed, the induced mass-action equations readily confirm this property, since

$$\frac{dX_1}{dt} = k_1 R - k_2 X_1 X_2, \tag{41}$$

$$\frac{dX_2}{dt} = k_3 - k_2 X_1 X_2, \tag{42}$$

and thus, it is clear by inspection that $\frac{dX_1}{dt} - \frac{dX_2}{dt} = k_1 R - k_3$. (So, $\alpha_1 = k_1$ and $\alpha_2 = k_3$ in this case.)

But note that *any* collection of chemical reactions could be embedded into the network at the indicated position in Supplementary Figure S7 - ie. outside of the opposer mechanism. These reactions could (and generally will) increase the deficiency of the network, possibly even making the deficiency very large ($\delta \gg 1$). That any such collection of reactions can necessarily be assigned to an algebraically independent subset is guaranteed by the fact that these reactions will all involve species that are not present in the deficiency-one antithetic integral controller subset (see Section S1.3). In any case, since these (potentially) deficiency-increasing chemical reactions belong to a different independent subset they do not change the setpoint ($R^* = \frac{k_3}{k_1}$), which is computed (as given above) by the deficiency-one antithetic integral controller subset.

Another clear example of deficiency-increasing chemical reactions that lie outside of the RPA-conferring subnetwork of a CRN was considered in Example 1 of the previous section

(see Supplementary Figure S8 below).

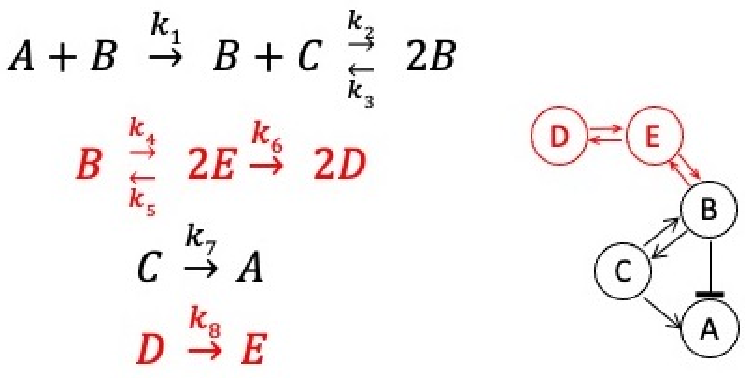

Supplementary Figure S8: Chemical reactions and topological structure for the toy model proposed by Cappelletti et al. [3], as examined in detail in Section S3.1 of the present Supplement.

In both of these examples (the antithetic integral control motif [2], and the toy model proposed by Cappelletti [3]), RPA is implemented by a deficiency-one subnetwork of the system. Any additional deficiency-increasing reactions have no bearing on the RPA capacity of the CRN, or the value of its setpoint, since these reactions belong to independent subnetworks of the CRN, distinct from the subnetwork that orchestrates the RPA property. Topologically, these reactions belong to extramodular contributions that are compatible with, but distinct from, the topological basis modules that engender RPA. In the case of Opposer modules, in particular, these additional deficiency-increasing reactions can also occur in the portion of the module that is distinct from the controller portion (i.e. the regulated or ‘controlled’ system).

S4.4 Deficiency-increasing reactions that contribute to RPA

A second possibility is for any deficiency exceeding unity to be embedded into the RPA-conferring reaction structures themselves, thereby contributing to the RPA mechanism. In contrast to the cases considered in the preceding section (S4.3), the deficiency-increasing reactions can no longer simply be discarded from analysis. Topologically, there are two distinct ways

this scenario can arise - one pertaining to Balancer modules, and one pertaining to Opposer modules.

S4.4.1 Balancer modules

If a CRN is topologically a Balancer module, it comprises at least two parallel pathways - at least one of which has an inhibitory (downregulating) effect on the connector variable, and at least one of which has a promoting (upregulating) effect. A balancer-type CRN with exactly two such parallel counteracting pathways (ie. the minimum), and without any additional deficiency-increasing reactions outside the parallel pathways (as was the case for the Cappelletti toy model in the previous section, for instance), will have a deficiency of *one*[1]. But an RPA-capable CRN with more than two parallel pathways in its balancer structure will increase its deficiency by one for each extra parallel pathway.

We demonstrate these principles through a detailed analysis of the deficiency-two EnvZ-OmpR motif studied by Shinar and Feinberg (see Supplementary Information to [16]), which we considered in Example 2 in Section S3.2. We reproduce the four linkage-class model of this deficiency-two CRN of the EnvZ-OmpR osmoregulation system in Supplementary Figure S9 below.

It is clear that the first two linkage classes, taken together, have defiency zero. But adding in *either* the third linkage class *or* the fourth linkage class increases the deficiency to one. The deficiency-one subnetwork formed by the first three linkage classes, for example, satisfies the Shinar-Feinberg theorem since there are now two non-terminal complexes - XT and $XT + Y_p$ - that differ in a single species (Y_p). Thus, these three linkage classes, taken together as a subnetwork, have the capacity for RPA (ACR) at Y_p. Likewise, the deficiency-one subnetwork

[1]The only exception to this is a 'trivial' Balancer module, which is simply a single connector node (no diverter or balancers), which therefore has deficiency-zero, Eg. $B+C \rightleftharpoons B$. It is easy to show that no such deficiency-zero RPA-capable CRN can be mass-conservative.

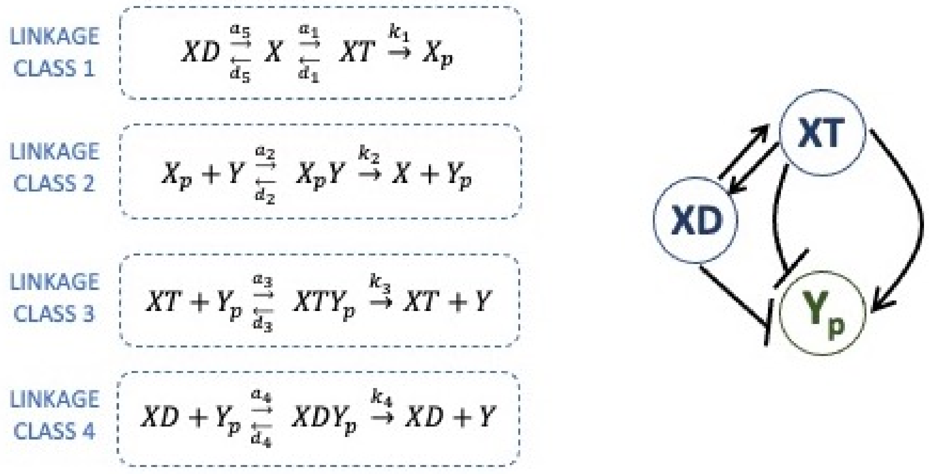

Supplementary Figure S9: Deficiency-two CRN of the EnvZ-OmpR osmoregulation system first studied by Shinar and Feinberg (see Supplementary Information to [16]).

formed from linkage classes 1, 2 and 4 also satisfies the Shinar-Feinberg theorem since there are two non-terminal complexes - this time, XD and $XD + Y_p$ - that differ in the species Y_p, thereby conferring RPA (ACR) on Y_p for this three linkage-class subnetwork.

It is clear from the topological structure of this CRN how additional linear dependencies of reactions (and hence higher deficiencies) arise as we successively add linkage classes to form larger subnetworks. Indeed, the first two linkage classes, taken together, contain the reactions (as differences between complexes, see [16]) $XT - X$, $X_p - XT$, $X_pY - Y - X_p$, and $X + Y_p - X_pY$. Adding these together gives $Y_p - Y$ in the span of the reactions, highlighting the fact that Y is converted to Y_p via these first two linkage classes. Furthermore, the third linkage class contains the reactions $XTY_p - XT - Y_p$ and $XT + Y - XTY_p$. Adding these together gives $Y - Y_p$, highlighting the fact that Y_p is converted back to Y by the reactions in this third linkage class. Likewise, the fourth linkage class contains the reactions $XDY_p - XD - Y_p$ and $XD + Y - XDY_p$, the sum of which produces $Y - Y_p$. Thus, the fourth linkage class has the same basic function as the third: to convert Y_p to Y.

To form a Balancer module, then, we require the first two linkage classes to be paired with *at least one* of linkage classes three or four. These latter two linkage classes provide two parallel pathways that counteract the pathway constructed by the first two linkage classes taken together. Indeed, for the deficiency-one subnetwork formed by the first three linkage classes, Theorem 3 guarantees that the following polynomials exist in the rowspan of the mass-action system corresponding to these three linkage classes in isolation:

$$\alpha_1 XT - \alpha_2 XT.Y_p, \tag{43}$$

$$\alpha_3 XT - \alpha_4 XD. \tag{44}$$

By contrast, for the deficiency-one subnetwork constructed from linkage classes 1, 2 and 4, Theorem 3 guarantees that the rowspan of the mass-action system corresponding to these three linkage classes will include the following polynomials:

$$\alpha_5 XT - \alpha_6 XD.Y_p, \tag{45}$$

$$\alpha_3 XT - \alpha_4 XD.$$

Thus, if all four linkage classes are now taken together, it follows that

$$\alpha_7 XT - \alpha_8 XT.Y_p - \alpha_9 XD.Y_p, \tag{46}$$

from polynomials (43) and (45) above, and

$$\alpha_3 XT - \alpha_4 XD,$$

will both reside in the rowspan of the full system. Polynomial (46) is the connector polynomial for the full system, whereas polynomial (44) is a balancer polynomial. As shown in Section S3.2, these two key polynomial invariants may be combined, via a concatenating monomial, to produce an RPA polynomial.

The connector polynomial (46) is a special case of *connector kinetics*, first identified in [1]. The general form of a connector polynomial is thus

$$m_1 X_c^{\beta} \pm \ldots \pm m_t X_c^{\beta} - m_{t+1} X_c^{\alpha} \ldots \pm m_T X_c^{\alpha}, \tag{47}$$

where $\beta \in \mathbb{Z}_{\geq 0}$, $\alpha \in \mathbb{Z}_{>0}$, and $\beta < \alpha$, and where $m_1 \ldots m_T$ are monomials, each of which involving one of the T *terminal* balancer molecules (corresponding to terminal balancer nodes, as given in [1]). In addition, X_c is the (potentially) RPA-capable variable of the CRN. Note that a diverter molecule may also be a terminal balancer molecule.

To achieve RPA, each terminal balancer monomial must be in direct proportion to the diverter variable at steady-state, and each such condition requires a corresponding balancer polynomial (e.g. polynomial (44)) to exist in the ideal associated to the CRN.

Although the collection of reactions we analyse here is of low deficiency ($\delta = 2$), with three parallel feedforward contributions, it is clear from the nature of the argument we propose above that this approach may be extended to Balancer-type CRNs of arbitrary deficiency (and thus, an arbitrary number of parallel feedforward contributions, with or without embedded feedback loops). Indeed, by an induction argument, starting with *any two* sufficient parallel pathways of the balancer module and adding in collections of chemical reactions corresponding to any additional parallel pathways (or embedded feedback loops if such exist), one pathway (or feedback loop) at a time, it is clear from Theorem 3 and the argument presented above, that the connector polynomial (47) can be constructed, one additional term at a time, along with all requisite balancer polynomials (e.g. polynomial (44)), and will *always* reside in the rowspan of the CRN's mass-action equations.

S4.4.2 Opposer modules

As we have noted previously, the simple antithetic integral control motif [2] (see Supplementary Figure S7), together with the network it controls (embedded into a feedback loop), is an example of an Opposer module [1]. As explained in Section S4.3, only the *controller reactions*, not the full network, need be considered in determining the RPA capacity and setpoint of the full network due to the associated partition into independent subnetworks. The antithetic integral controller itself is an example of a *deficiency-one* CRN of opposer type, whose mass action equations contain an RPA polynomial in its rowspan that encodes *opposer kinetics* [1].

Neglecting the 'unnecessary' (and potentially deficiency increasing) reactions in the embedded network (i.e. outside of the controller reactions), the controller CRN of an Opposer module could still increase its deficiency beyond one due to the presence of multiple distinct, but interconnected, feedback loops which together orchestrate RPA. Recall that each addition of a collection of reactions that 'replicates' an existing collection of reactions, in such a way as to create feedback within the CRN, results in a deficiency increase of one. But where multiple distinct feedback loops are involved, these CRNs can be decomposed into multiple independent subsets. Each independent subset will thus contribute its own subsidiary polynomial invariant, which can be computed readily using the approach we developed in the previous section. Topologically, this scenario corresponds to a structure known as an *opposing set* [1].

We illustrate how this type of RPA-conferring controller structure can be partitioned into independent CRN subsets, each contributing an RPA-relevant invariant within the rowspan of the system (which can subsequently be combined to yield the all-important RPA polynomial) through the analysis of the example given in Figure 2b in the main paper. We present this network organised into its correct linkage classes in Supplementary Figure S10. This CRN has a deficiency of three, since it comprises ten complexes ($m = 10$), three linkage classes ($l = 3$), and has a rank of four ($s = 4$). The three 'units' of deficiency correspond to the presence of the

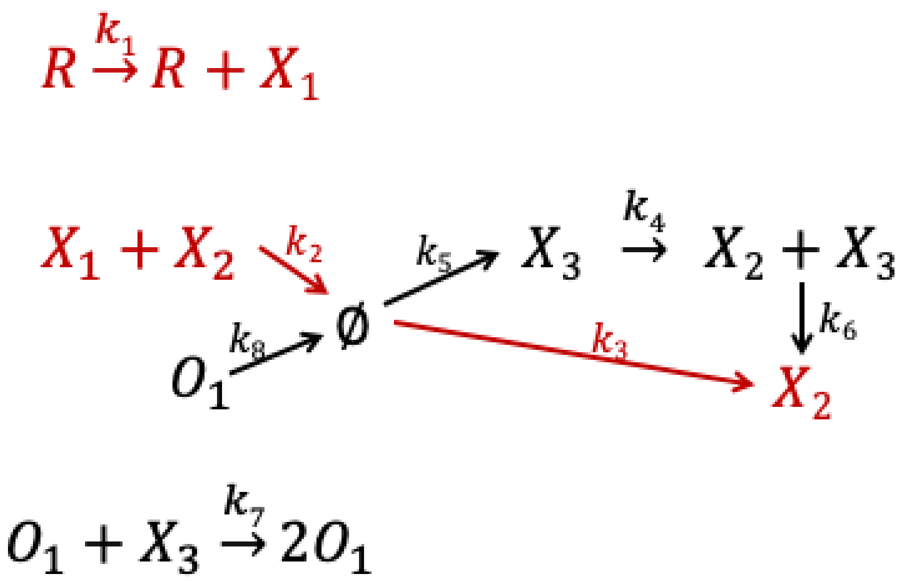

Supplementary Figure S10: CRN presented in Figure 2b in the main paper, organised into linkage classes, and employing the antithetic integral control motif [2] as one of two opposers in a two-node opposing set.

three interlinked feedback loops that comprise a two-node opposing set:

1. The reaction $R + X_1 - R = X_1 - \emptyset$ (at rate k_1) is replicated by the linear combination $(\emptyset - X_1 - X_2)$ + $(X_2 - \emptyset)$, at rates k_2 and k_3 respectively. This is the 'opposer cycle', regulated by R, that ultimately confers RPA on R.

2. The reaction $X_2 - X_3$ (from the linear combination $(X_2 - X_2 - X_3)$ + $(X_3 + X_2 - X_3)$, at rates k_6 and k_4, respectively) is replicated by $(X_2 - \emptyset)$ - $(X_3 - \emptyset)$, at rates k_3 and k_5, respectively. This is the intermediate cycle interlinking the two 'opposer' cycles, as required for an opposing set structure (see [1]).

3. The reaction $O_1 - X_3$ (at rate k_7) is replicated by the combination $(X_3 - \emptyset)$ - $(\emptyset - O_1)$, at rates k_5 and k_8 respectively. This is the second (distal) opposer cycle, which imposes RPA on X_3.

In Supplementary Figure S11, we decompose this CRN into two independent subnetworks, each with a rank of two ($s = 2$). Each of these subnetworks has a deficiency of two. Since the two subnetworks are independent, they can be analysed separately with respect to the polynomial invariants in their respective rowspans.

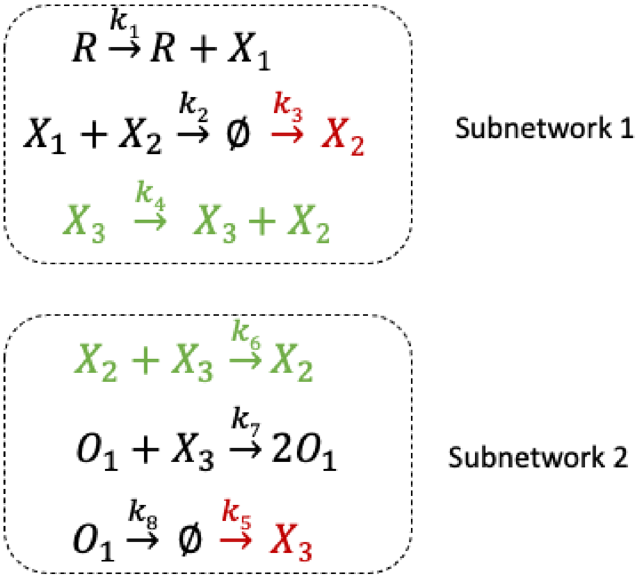

Supplementary Figure S11: CRN presented in Supplementary Figure S10, decomposed into two independent subnetworks, each with a rank, $s = 2$.

Consider Subnetwork 1. If *either* the reaction noted in red *or* the reaction noted in green is omitted, the deficiency of the remaining subnetwork reduces to one. In particular, neglecting the green reaction, the subnetwork reduces to the antithetical control motif, and contains an RPA polynomial of the form $\alpha_1 R - \alpha_2$ in its rowspan as a consequence of Theorem 3. If, instead, the red reaction is neglected, the remaining deficiency-one network contains a polynomial of the form $\alpha_3 R - \alpha_4 X_3$ in its rowspan. It follows that if *both* the red *and* the green reactions are included, a polynomial of the form $\alpha_5 R - \alpha_6 - \alpha_7 X_3$ is present in the rowspan of the mass-action equations associated to Subnetwork 1. This is no longer an RPA polynomial. Indeed, as we note in the main paper, the inclusion of the green reaction in this subnetwork prevents

this CRN from achieving RPA by itself, and requires an additional controller structure (in this example, Subnetwork 2) in order to preserve the RPA-promoting properties of the antithetic integral control motif.

Consider Subnetwork 2. Here again, if *either* the reaction noted in red *or* the reaction noted in green is omitted, the deficiency of the remaining subnetwork reduces to one. In either case, the deficiency-one subnetwork contains a polynomial of the form $\alpha_8 O_1 X_3 - \alpha_9 O_1$ - an RPA polynomial for X_3 - as a consequence of Theorem 3. Therefore, the full Subnetwork 2 also contains a polynomial $\alpha_8 O_1 X_3 - \alpha_9 O_1$ in its rowspan, and confers the capacity for RPA on X_3. This being the case, this invariant can now be 'passed' to the invariant $\alpha_5 R - \alpha_6 - \alpha_7 X_3$ for Subnetwork 1, which can now be interpreted as an opposer invariant. As indicated in Figure2c of the main paper, combining the opposer invariants from each of the two independent subnetworks requires the *concatenating monomial* O_1 to be applied to the opposer polynomial from Subnetwork 1 (since this contains the monomial X_3) in order to combine with the opposer polynomial from Subnetwork 2 (since this contains the monomial $O_1 X_3$), in order to produce the RPA polynomial given in Figure 2c of the main paper.

The CRN presented in Figure 2c of the main paper, decomposed into two independent subnetworks in Supplementary Figure S11, is thus a *two-node opposing set*, since it contains two independent *opposer invariants* (or *opposer polynomials*), each contained in the rowspan of the CRN's mass action equations:

$$\alpha_5 R - \alpha_6 - \alpha_7 X_3, \tag{48}$$

$$\alpha_8 O_1 X_3 - \alpha_9 O_1, \tag{49}$$

where it is clear that, for this example, $\alpha_5 = k_1$, $\alpha_6 = k_3$, $\alpha_7 = k_4$, $\alpha_8 = k_7$ and $\alpha_9 = k_8$. The topological structures of *opposing sets* are now well understood, and are fully described in

[1]. From this point of view, we consider polynomial (48) to be the *proximal* opposer invariant, since it is directly regulated by the embedded network (which exhibits RPA, and which is not explicitly considered in the analysis of the RPA capacity of the Opposer module as a whole). From a control theory viewpoint, the molecule R could be considered the *sensor molecule*. The polynomial (49), on the other hand, is the most *distal* opposer invariant, being the polynomial furthest away (topologically speaking) from the sensor molecule, and whose non-RPA exhibiting molecule (here O_1) - which must regulate the embedded network in order for the system as a whole to have the capacity for RPA - could be considered an *actuator molecule* from a control theory viewpoint.

The principles considered above for a particular two-node opposing set can thus readily be generalised to any controller CRN of an Opposer module, with arbitrary numbers of independent opposer polynomials, and thus arbitrarily high deficiency, as predicted in the general theory of RPA at the network macroscale given in [1]. Owing to the topological principles governing opposing sets, any controller CRN for an Opposer module can be decomposed into n independent subnetworks ($n \geq 1$), with each such subnetwork contributing one opposer polynomial ρ_i which exists in the *rowspan* of the mass action equations of the respective subnetwork. The distal opposer polynomial, will take the form

$$\rho_n = g(x_{jn})(x_{in} - c_n), \tag{50}$$

where x_{jn} is an 'actuator molecule' (i.e. non RPA-capable) for the distal subnetwork, x_{in} is an RPA-capable molecule in the distal subnetwork, and c_n is the setpoint of x_{in} (a rational function of rate constants in the distal subnetwork). For the remaining $n - 1$ independent subnetworks, the opposer polynomial will take the form

$$\rho_k = g(x_{jk})(x_{ik} - c_k - \hat{c}_k p_{k+1}), \tag{51}$$

where p_{k+1} is a polynomial involving RPA-capable molecule(s) from the $(k+1)$-th (i.e. next most distal) subnetwork that also participate in subnetwork k, and where c_k and $\hat{c}_k$ are rational functions of rate constants in subnetwork k. The setpoint for the RPA-capable molecule x_{ik} is thus $c_k + \hat{c}_k c_{k+1}$.

S4.5 A note on non-RPA-capable CRNs and computational challenges

As noted throughout this study, the 'almost linear' nature of the mathematical transformation that decomposes the RPA polynomial into a connected collection of linear integral controllers guarantees that *for an RPA-capable CRN*, the Gröbner-basis computing algorithm we describe here, for judicious choice of two variables, will terminate in polynomial time. But for the study of general CRNs, which may be large and highly complicated, the RPA capacity or otherwise of a particular CRN may be entirely unclear if the algorithm appears to require a long time to terminate.

Here we briefly review again the analytical approaches that can be used to check whether a CRN at hand has any potential for RPA capacity through an analysis of deficiency (and a prior decomposition into independent subnetworks, wherever possible). Nevertheless, it's worth always bearing in mind that one should have a reason to suspect that a CRN exhibits RPA. It is clear from our analysis of RPA-capable CRNs at the network microscale, and from our topological analysis of RPA-capable network architectures at the network macroscale [1], that RPA-capable networks are actually very 'special', with very specific structural requirements, and are therefore extremely rare in the space of all possible networks. If one has a completely arbitrary network, with no particular reason to suspect it might be able to exhibit this special type of robustness, then it almost certainly doesn't.

By way of example, consider the CRN for the mammalian enzyme 6-phosphofructo-2-kinase/fructose-2,6-bisphosphatase (*PFK-2/FBPase-2*), which operates bifunctionally to both

activate and inactivate fructose-2,6-bisphosphate (*F2,6BP*), as discussed by Karp et al. [12] in terms of the potential RPA-capacity of *F2,6BP*. By virtue of involving a bifunctional enzyme, as was the case for the Shinar-Feinberg model [16] of the EnvZ-OmpR motif, one might suspect that this network could exhibit RPA (or ACR, more specifically) in *F2,6BP*. But as we will show, this CRN *cannot* exhibit RPA in *F2,6BP* or any other molecule. The CRN (presented in [12]), organized into its linkage classes, with $X_1 \equiv$ *E*, $X_2 \equiv$ *E-ATP*, $X_3 \equiv$ *F6P*, $X_4 \equiv$ *E-ATP-F6P*, $X_5 \equiv$ *F2,6BP*, $X_6 \equiv$ *E- F2,6BP*, $X_7 \equiv$ *E-ATP-F2,6BP*, and $X_8 \equiv$ *E-ATP-F6P-F2,6BP*, is depicted in Supplementary Figure S12.

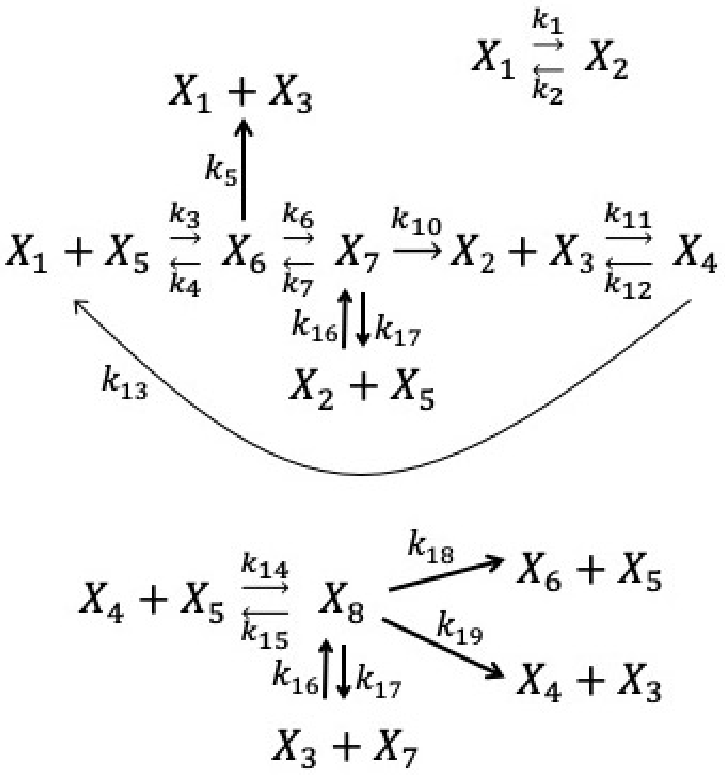

Supplementary Figure S12: CRN for the mammalian enzyme 6-phosphofructo-2-kinase/fructose-2,6-bisphosphatase (*PFK-2/FBPase-2*). For full details of the CRN, see Karp et al. [12].

For a CRN as simple as this, with only eight variables, we can apply Theorem 1 via computation of a suitable Gröbner-basis. Interestingly, Karp et al.[12] claim that the Gröbner basis

implementation in Mathematica does not terminate for this CRN, although they give no information as to how they ordered their variables, or which monomial ordering they chose (although they almost certainly would have used a lexicographic monomial ordering since, prior to the present study, it was not known that an elimination ideal involving just two variables is all that's required to test RPA capacity; the lexicographic ordering is an extremely computationally expensive elimination ordering, in general, and it produces a full complement of elimination ideals - in vast excess of what's actually required to solve the RPA problem). We used Singular, and used an efficient block monomial ordering (to project onto two variables) that allowed the algorithm to terminate in seconds, from which we could confirm that this particular CRN is not RPA-capable (at $X_5 =$ *F2,6BP* or otherwise). Our Singular code listing is given in Section S5.5.

Upon careful scrutiny of the resulting Gröbner basis it is evident that, for this CRN, $I_f \cap \mathbb{R}[X_1, X_5]$ contains only zero. Thus, this CRN is not RPA-capable (at X_5). It may readily be shown by this method that RPA does not obtain for any variable of the CRN.

While this simple non-RPA-capable CRN in eight variables yields easily to a suitable Gröbner basis computation, it is certainly conceivable that vastly larger and more complicated CRNs could require a significantly longer timeframe for the algorithm to terminate. By way of general advice to the reader, if one allows the algorithm to run for several hours (e.g. overnight) using the most efficient implementation possible (as described above; see also our code listing in Section S5.5) and it still hasn't terminated after this period, then one should consider undertaking some additional analysis of the CRN (as we provide below) through (i) first decomposing into algebraically-independent subnetworks (whenever possible), and then (ii) considering where deficiency arises within these independent subnetworks, and whether the deficiency corresponds to the presence of parallel pathways and/or feedback cycles, as required for RPA.

Now, recall that deficiency is a measure of the linear independence of the reactions of a

CRN, relative to their distribution into the connected components (linkage classes) of a graph. In particular, the deficiency of a CRN is increased by one for every instance of a reaction being 'replicated' elsewhere in the network via a different set of complexes. Reversible reactions, for example, which duplicate a single reaction using the same pair of complexes, do not of themselves contribute to any deficiency increases. Recall also that, for an RPA-capable CRN (or subnetwork), these replicated reactions must be able to represent either (i) a collection of parallel pathways, where the production/degradation (or interconversion between activation states) of the RPA-molecule must be orchestrated via different collections of reactions (involving different sets of complexes), or (ii) a cycle in the production/degradation (or interconversion between activation states) of a non-RPA-molecule (as regulated by the RPA-capable molecule), where the individual components of the cycle must be orchestrated via different collections of reactions (involving different sets of complexes). We have highlighted these fundamental requirements throughout the analysis of all illustrative examples in this Supplement; there's simply no other way for the requisite linear invariants to emerge!

Now, the CRN at hand has a deficiency of 5, comprising 14 complexes, 3 linkage classes and a rank of 6. By noting which reactions are 'replicated' (using different sets of complexes) to yield each of these 5 units of deficiency, it is straightforward to see that this CRN does not have the right structure to impose RPA on X_5 (or any other molecule). First, we note that the eight species of the model are all intricately interconnected in the nineteen reactions of the CRN, such that no decomposition into independent subsets is possible (see discussion of this point in Section S1.3). Moreover, in assessing the linear independence of the CRN reactions, only one reaction in each pair of reversible reactions need be considered (for the reasons noted above pertaining to the deficiency-preserving nature of reversible reactions). Now, from a routine linear-algebraic analysis of the remaining reactions, it is easy to show that the reactions occurring at rates $k_1, k_3, k_5, k_6, k_{11}$ and k_{14} form a linearly independent set that spans the sto-

ichiometric subspace of this CRN. The linear dependencies that give rise to the five units of deficiency are (with rate constants noted by underbraces to highlight the identity of the reaction in question):

$$\underbrace{X_7 - X_5 - X_2}_{k_8} = -\underbrace{(X_2 - X_1)}_{k_1} + \underbrace{X_6 - X_1 - X_5}_{k_3} + \underbrace{X_7 - X_6}_{k_6}, \tag{52}$$

$$\underbrace{X_3 + X_2 - X_7}_{k_{10}} = \underbrace{X_2 - X_1}_{k_1} + \underbrace{X_1 + X_3 - X_6}_{k_5} - \underbrace{(X_7 - X_6)}_{k_6}, \tag{53}$$

$$\underbrace{X_8 - X_7 - X_3}_{k_{16}} = \underbrace{X_2 - X_1}_{k_1} - \underbrace{(X_6 - X_1 - X_5)}_{k_3} - \underbrace{(X_7 - X_6)}_{k_6} + \underbrace{X_4 - X_2 - X_3}_{k_{11}} + \underbrace{X_8 - X_4 - X_5}_{k_{14}}, \tag{54}$$

$$\underbrace{X_5 + X_6 - X_8}_{k_{18}} = -\underbrace{(X_2 - X_1)}_{k_1} - \underbrace{(X_1 + X_3 - X_6)}_{k_5} - \underbrace{(X_4 - X_2 - X_3)}_{k_{11}} - \underbrace{(X_8 - X_4 - X_5)}_{k_{14}}, \tag{55}$$

$$\underbrace{X_3 + X_4 - X_8}_{k_{19}} = \underbrace{X_6 - X_1 - X_5}_{k_3} + \underbrace{X_1 + X_3 - X_6}_{k_5} - \underbrace{(X_8 - X_4 - X_5)}_{k_{14}}. \tag{56}$$

It is clear that these five distinct replicated reactions have no relationship whatsoever to either a collection of parallel pathways, to form a balancer module, or a cycle (or collection of interconnected cycles corresponding to an opposing set), to form an opposer module. None of these replicated reactions captures the production or degradation of a particular molecule, nor the interconversion between any one molecule and another molecule. This analysis thereby constitutes an independent confirmation that this CRN is not RPA-capable (at X_5 or otherwise).

S4.6 Summary of general principles for RPA in CRNs

The following represents a summary of the general principles governing the RPA capacity of all CRNs, as outlined in the preceding sections:

1. For each RPA-promoting module, the key subsidiary polynomial invariants reside in the

rowspan of the CRN. In particular,

(a) For a Balancer module, the connector polynomial, along with all requisite balancer polynomials, reside in the rowspan of the CRN.

(b) For an Opposer module, comprising an n-node opposing set ($n \geq 1$), each of the n opposer polynomials resides in the rowspan of the CRN.

2. Large and complex RPA-capable CRNs could be composed of multiple such modules, connected together. The full set of topological possibilities for how this may occur is now understood in complete generality (see [1]). It is clear from the analysis in the preceding sections that a CRN can be decomposed into lower deficiency independent subnetworks corresponding to the individual modules of a multi-modular network and, in the case of Opposer modules, the controller portion of the module, which can be analysed separately.

3. For Balancer modules, invariants are 'passed' downstream from the diverter to each terminal balancer, culminating in the construction of an RPA polynomial from the connector polynomial. By contrast, for Opposer modules, invariants are passed from the distal opposer to the proximal opposer.

4. If the subsidiary polynomials (balancers, connectors, opposers) are *stoichiometrically independent* (see Section S3), they require a concatenating monomial to combine invariants to ultimately produce the RPA polynomial. Otherwise, they can combine without leaving the rowspan of the CRN's mass-action equations. Thus, in special cases, the RPA polynomial itself may reside in the rowspan. (CRNs satisfying the Shinar-Feinberg theorem are included among these special cases.)

5. In all cases, the fact that *all* the key invariants of all RPA-capable CRNs exist in the rowspan of the mass-action equation, reveals a well-defined connection to integral control, as

outlined in the main paper.

The analysis in this Section (S4) makes clear that sets of polynomials $\{h_1, \ldots, h_n\}$ that are able to eliminate all but two variables from the rate equations to 'reach' the RPA polynomial of the CRN (Theorem 1) possess a very special structure. In particular,

$$[h_1, \ldots, h_n]^T \in Z \oplus \hat{h}, \tag{57}$$

where Z is the module of syzygies of the CRN rate equations, and $\hat{h}$ is the 'canonical' basis set of polynomials $h_1, \ldots, h_n$ that 'reaches' the RPA polynomial of the CRN.

Some CRNs will contain mass-conservation equations. For these, the module of syzygies may be further decomposed into the form

$$Z \oplus \hat{h} = Z_x \oplus Z_\phi \oplus \hat{h}, \tag{58}$$

where Z_ϕ is the co-kernel (left nullspace) of $Y.\mathcal{L}(G)$. CRNs without mass-conservation relations will have a trivial co-kernel, with $Z = Z_x$. In any case, it follows from the analysis earlier in this Section that the only monomials contained in any polynomial in $Z_\phi \oplus \hat{h}$, for judicious choice of two projection variables (see Section S2), are the requisite concatenating monomials.

Given this special 'almost linear' structure of the ideal associated to an RPA polynomial, RPA capacity may be tested systematically via Buchberger's algorithm, which computes the Gröbner basis of an ideal, with an elimination ordering on monomials. In addition, using this algorithm, a set of elimination polynomials $\{h_1, \ldots, h_n\} \in Z_\phi \oplus \hat{h}$ can be computed automatically. In the next Section, we provide code to make these systematic computations in the open-source software *Singular* (www.singular.uni-kl.de) [5], which can be adapted to the study of any CRN.

S5 Singular code for analysing the RPA capacity of CRNs

S5.1 General structure of Singular code

The general structure of a *Singular* script that systematically tests the RPA capacity of a CRN and automatically determines the subsidiary polynomial invariants along with a set of elimination polynomials, $\hat{h}$, involves the following commands:

1.
```
> ring R = (0, {parameters listed in any order}),
     (variables listed with two projection variables LAST), lp;
```

   Note that the '0' noted before the parameter list denotes the characteristic of the field. For the analysis of CRNs, the relevant underlying field is the real numbers, which has characteristic zero. The command 'lp' denotes *lexicographic order*, which is an elimination order on the monomials of the system. The command 'lp' can be always be replaced with the block (elimination) order '(dp($n-2$), dp(2))', which imposes the much more computationally efficient *degree reverse lexicographic order* on both the block of $(n-2)$ variables to be eliminated and the block of 2 variables that remain. But as we note, the special structure of RPA-capable CRNs allows the Gröbner basis to be computed in polynomial time for these networks, such that the conceptually simpler (albeit computationally more expensive) lexicographic ordering will generally perform adequately if RPA does indeed obtain.

2. Next, the n mass-action rate equations are listed in any order.

```
> poly f1 = ... ;

    ⋮

> poly fn = ... ;
```

3. We can now compute the ideal generated by the rate equations $f_1, \ldots, f_n$:

```
> ideal I = f1, ... , fn;
```

4. Finally, we can compute the Gröbner basis for the ideal with the chosen monomial ordering:

```
> ideal GI = groebner(I);
> GI;
```

   For the CRN to be RPA-capable, only the first polynomial in the Gröbner basis (listed in Singular as $GI[1]$) can comprise only the two projection variables, and it must be factorizable into an RPA polynomial.

5. We can now automatically calculate the subsidiary polynomial invariants, as well as a set of polynomials $h_1, \ldots, h_n$ for which $h_1 f_1 + \ldots + h_n f_n = \rho = GI[1]$ via Singular's *lift* command:

```
> lift(I, GI[1]);
```

6. The presence of any conservation laws in the system can also be quickly checked by calculating the syzygies of the system via the *syz* command:

```
syz(I);
```

   If conservation laws do exist for the system in question, the module of syzygies will contain generators (one corresponding to each conservation law) containing only integer elements.

We demonstrate the application of this Singular code through a listing of scripts used to analyse the illustrative examples featured in this work.

S5.2 Singular code for Example in Section S1.6 (Figure 2, main paper)

```
> ring R1 = (0, k1, k2, k3, k4, k5, k6, k7, k8), (x3, x1, o1, x2, r), lp;
> poly f1 = k1*r - k2*x1*x2;
> poly f2 = k3 - k2*x1*x2 + k4*x3;
> poly f3 = k5 - k7*o1*x3 - k6*x2*x3;
> poly f4 = k7*o1*x3 - k8*o1;
> ideal I = f1, f2, f3, f4;
> ideal GI = groebner(I);
> GI;
```

This code projects the system onto the two variables $x2$ and r, and generates the following output:

$$
\begin{aligned}
GI[1] = {} & (k1^2 * k6 * k7) * x2 * r^2 + (-2 * k1 * k3 * k6 * k7 - k1 * k4 * k6 * k8) * x2 * r \\
& + (k3^2 * k6 * k7 + k3 * k4 * k6 * k8) * x2 + (-k1 * k4 * k5 * k7) * r \\
& + (k3 * k4 * k5 * k7 + k4^2 * k5 * k8)
\end{aligned}
$$

$$
GI[2] = (k4 * k8) * o1 + (k1 * k6) * x2 * r + (-k3 * k6) * x2 + (-k4 * k5)
$$

$$
\begin{aligned}
GI[3] = {} & (k1 * k2 * k4 * k5 * k7) * x1 * r + (-k2 * k3 * k4 * k5 * k7 - k2 * k4^2 * k5 * k8) * x1 \\
& + (-k1^3 * k6 * k7) * r^3 + (2 * k1^2 * k3 * k6 * k7 + k1^2 * k4 * k6 * k8) * r^2 \\
& + (-k1 * k3^2 * k6 * k7 - k1 * k3 * k4 * k6 * k8) * r
\end{aligned}
$$

$$
GI[4] = (k2) * x1 * x2 + (-k1) * r
$$

$$
GI[5] = (k4) * x3 + (-k2) * x1 * x2 + (k3)
$$

As shown in-text, G[1] has the form of an RPA polynomial. We can also repeat this process for the range of alternative projections noted in Section S1.6. For instance, projecting onto $o1$ and

r requires the code:

```
> ring R2 = (0, k1, k2, k3, k4, k5, k6, k7, k8), (x3, x1, x2, o1, r), lp;
> poly f1 = k1*r - k2*x1*x2;
> poly f2 = k3 - k2*x1*x2 + k4*x3;
> poly f3 = k5 - k7*o1*x3 - k6*x2*x3;
> poly f4 = k7*o1*x3 - k8*o1;
> ideal I = f1, f2, f3, f4;
> ideal GI = groebner(I);
> GI;
```

which generates the following output in Singular:

$$GI[1] = (k1 * k7) * o1 * r + (-k3 * k7 - k4 * k8) * o1$$

$$GI[2] = (k1 * k6) * x2 * r + (-k3 * k6) * x2 + (k4 * k8) * o1 + (-k4 * k5)$$

$$GI[3] = (k6 * k8) * x2 * o1 + (k7 * k8) * o1^2 + (-k5 * k7) * o1$$

$$GI[4] = (k1 * k2 * k4 * k5 * k7) * x1 * r + (-k2 * k3 * k4 * k5 * k7 - k2 * k4^2 * k5 * k8) * x1$$
$$+ (-k1^3 * k6 * k7) * r^3 + (2 * k1^2 * k3 * k6 * k7 + k1^2 * k4 * k6 * k8) * r^2$$
$$+ (-k1 * k3^2 * k6 * k7 - k1 * k3 * k4 * k6 * k8) * r$$

$$GI[5] = (k2 * k4 * k8) * x1 * o1 + (-k2 * k4 * k5) * x1 + (k1^2 * k6) * r^2 + (-k1 * k3 * k6) * r$$

$$GI[6] = (k2) * x1 * x2 + (-k1) * r$$

$$GI[7] = (k4) * x3 + (-k2) * x1 * x2 + (k3)$$

From this it is clear by inspection that $GI[1]$ is an RPA polynomial. The subsidiary polynomial invariants and elimination polynomials may now be calculated using the *lift* command:

```
> lift(I, G[1]);
```

which returns the following output:

$$[1,1] = (k7) * o1$$
$$[2,1] = (-k7) * o1$$
$$[3,1] = 0$$
$$[4,1] = (k4)$$

This reveals that

$$G[1] = k7.o1.f1 - k7.o1.f2 + k4.f4,$$
$$= k7.o1(f1 - f2) + k4(f4),$$

which demonstrates that one concatenating monomial, $o1$, is required, and that the two subsidiary polynomial invariants (opposer polynomials) are $f_1 - f_2 = k_1.r - k_3 - k_4.x_3$ and $f_4 = k_7.o_1.x_3 - k_8.o_1$. The command *syz* reveals that this system has no non-trivial left nullspace (co-kernel). These two linear transformations are thus the canonical transformations for identifying the two subsidiary polynomial invariants.

S5.3 Singular code for Example in Section S3.1 (Cappelletti et al. [3] toy model)

```
> ring R = (0, k1,k2,k3,k4,k5,k6,k7,k8), (E,D,C,B,A), lp;
> poly f1 = -k1*A*B + k7*C;
> poly f2 = k2*B*C - k3*B^2 + k5*E^2 -k4*B;
> poly f3 = k1*A*B -k7*C - k2*B*C + k3*B^2;
> poly f4 = 2*k6*E^2 - k8*D;
```

```
> poly f5 = k8*D + 2*k4*B - 2*k5*E^2 - 2*k6*E^2;
> ideal I = f1, f2, f3, f4, f5;
> ideal GI = groebner(I);
> GI;
```

Running this code in Singular generates the following output:

$$GI[1] = (k1 * k2) * B^2 * A + (-k3 * k7) * B^2$$

$$GI[2] = (k7) * C + (-k1) * B * A$$

$$GI[3] = (k5 * k8) * D + (-2 * k4 * k6) * B$$

$$GI[4] = (2 * k6) * E^2 + (-k8) * D$$

from which it is clear that $GI[1]$ is an RPA polynomial. Using the *lift* command now calculates the subsidiary polynomial invariants and elimination polynomials:

```
> lift(I, G[1]);
```

which returns the following output:

$$[1, 1] = (-k2) * B$$

$$[2, 1] = (k7)$$

$$[3, 1] = 0$$

$$[4, 1] = (k7)/2$$

$$[5, 1] = (k7)/2$$

This demonstrates that one concatenating monomial, B, is required, and that the two subsidiary polynomial invariants (opposer polynomials) are $f_1 = -k_1.A.B+k_7.C$ (the connector invariant) and $k_7(f_2 + (1/2)f_4 + (1/2)f_5) = k_2.B.C - k_3.B^2$ (the balancer invariant).

The command *syz* reveals that this system has one conservation law, with

```
> syz(I);
```

producing an output in which only one generator contains integer elements:

$$[1] = gen(5) + gen(4) + 2 * gen(3) + 2 * gen(2) + 2 * gen(1)$$

This demonstrates that, for this CRN, $E + D + 2C + 2B + 2A$ is a constant for all time. Thus, the linear coordinate changes noted above to produce the balancer/connector invariants are not unique. But with the connector and balancer invariants automatically calculated by this algorithm, the canonical linear transformation can quickly be determined by routine calculation by the methods developed by [12, 3].

S5.4 Singular code for Example in Section S3.2 (EnvZ-OmpR Osmoregulation, Figure 3, main paper)

```
> ring R = (0, a1, d1, k1, a2, d2, k2, a3, d3, k3, a4, d4, k4, a5, d5),
                  (x9, x8, x7, x6, x5, x4, x3, x2, x1), lp;
> poly f1 = a1*x4 - (d1+k1)*x1 - a3*x1*x2 + (d3+k3)*x8;
> poly f2 = k2*x7 - a3*x1*x2 + d3*x8 - a4*x3*x2 + d4*x9;
> poly f3 = d5*x4 - a5*x3 - a4*x3*x2 + (k4+d4)*x9;
> poly f4 = a5*x3 + d1*x1 - (a1+d5)*x4 + k2*x7;
> poly f5 = k1*x1 - a2*x5*x6 + d2*x7;
> poly f6 = d2*x7 - a2*x5*x6 + k3*x8 + k4*x9;
> poly f7 = a2*x5*x6 - (d2+k2)*x7;
> poly f8 = a3*x1*x2 - (d3+k3)*x8;
> poly f9 = a4*x2*x3 - (d4+k4)*x9;
> ideal I = f1, f2, f3, f4, f5, f6, f7, f8, f9;
```

```
> ideal GI = groebner(I);
> GI;
```

Running this code in Singular generates the following output:

$$GI[1] = (a1*a3*k3*d4*a5 + a1*a3*k3*k4*a5 + d1*d3*a4*k4*d5 + d1*k3*a4*k4*d5 + k1*d3*a4*k4*d5 + k1*k3*a4*k4*d5)*x2*x1 + (-a1*k1*d3*d4*a5 - a1*k1*d3*k4*a5 - a1*k1*k3*d4*a5 - a1*k1*k3*k4*a5)*x1$$

$$GI[2] = (a1*a5)*x3 + (-d1*d5 - k1*d5)*x1$$

$$GI[3] = (d5)*x4 + (-a5)*x3$$

$$GI[4] = (a2*k2)*x6*x5 + (-a1*d2 - a1*k2 - d2*d5 - k2*d5)*x4 + (d2*a5 + k2*a5)*x3 + (d1*d2 + d1*k2)*x1$$

$$GI[5] = (k2)*x7 + (-a1 - d5)*x4 + (a5)*x3 + (d1)*x1$$

$$GI[6] = (d3 + k3)*x8 + (-a3)*x2*x1$$

$$GI[7] = (k4)*x9 + (k3)*x8 + (-k1)*x1$$

It is clear that $G[1]$ is an RPA polynomial. In addition running the *lift* command,

```
> lift(I, GI[1]);
```

produces the following output:

$[1,1] = (-a1*d3*a4*k4 - a1*k3*a4*k4 - d3*a4*k4*d5 - k3*a4*k4*d5)*x2$

$[2,1] = 0$

$[3,1] = 0$

$[4,1] = (-a1*d3*a4*k4 - a1*k3*a4*k4)*x2$

$[5,1] = (-a1*d3*a4*k4 - a1*k3*a4*k4)*x2$

$+ (-a1*d3*d4*a5 - a1*d3*k4*a5 - a1*k3*d4*a5 - a1*k3*k4*a5)$

$[6,1] = (a1*d3*d4*a5 + a1*d3*k4*a5 + a1*k3*d4*a5 + a1*k3*k4*a5)$

$[7,1] = (-a1*d3*a4*k4 - a1*k3*a4*k4)*x2$

$[8,1] = (-a1*d3*a4*k4 - a1*k3*a4*k4 - d3*a4*k4*d5 - k3*a4*k4*d5)*x2$

$+ (a1*k3*d4*a5 + a1*k3*k4*a5)$

$[9,1] = (a1*d3*k4*a5 + a1*k3*k4*a5)$

Thus, only one concatenating monomial $x2$ is required, and there are two subsidiary polynomial invariants, namely

$$
\begin{aligned}
&-\alpha f_5 + \alpha f_6 + \beta f_8 + \gamma f_9 = (a1*d3*a4*k4*a5 + a1*k3*a4*k4*a5)*x3*x2 \\
&+ (a1*a3*k3*d4*a5 + a1*a3*k3*k4*a5)*x2*x1 \\
&+ (-a1*k1*d3*d4*a5 - a1*k1*d3*k4*a5 - a1*k1*k3*d4*a5 - a1*k1*k3*k4*a5)*x1,
\end{aligned}
$$

being the connector invariant, with $\alpha = (a1*d3*d4*a5 + a1*d3*k4*a5 + a1*k3*d4*a5 + a1*k3*k4*a5)$, $\beta = (a1*k3*d4*a5 + a1*k3*k4*a5)$, and $\gamma = (a1*d3*k4*a5 + a1*k3*k4*a5)$;

and

$$(\eta+\zeta)(f_1+f_8)+\eta(f_4+f_5+f_7) = (-a1*d3*a4*k4*a5 - a1*k3*a4*k4*a5)*x3$$
$$+ (d1*d3*a4*k4*d5 + d1*k3*a4*k4*d5 + k1*d3*a4*k4*d5 + k1*k3*a4*k4*d5)*x1,$$

being the balancer invariant, with $\eta = -a1*d3*a4*k4 - a1*k3*a4*k4$, and $\zeta = -d3*a4*k4*d5 - k3*a4*k4*d5$.

The command,

```
> syz(I);
```

produces an output for which only two generators contain integer elements:

$$[1] = gen(6) - gen(5) - gen(4) - gen(3) + gen(2) - gen(1)$$
$$[2] = gen(9) + gen(8) + gen(7) + gen(5) + gen(4) + gen(3) + gen(1)$$

This confirms that there are two conservation laws, with $x_9+x_8+x_7+x_5+x_4+x_3+x_1$ equal to a constant for all time (conserving the total abundance of X), and $x_6+x_2-(x_5+x_4+x_3+x_1) = x_6+x_2+x_9+x_8+x_7$ equal to a constant for all time (conserving the total abundance of Y). Thus, once again, the linear coordinate changes by which the connector and balancer invariants above are obtained are not unique. But having systematically calculated these two subsidiary invariants as shown above, the canonical linear transformation can quickly be determined by routine calculation by the methods developed by [12, 3]. It is this canonical transformation that we depict in Figure 2c of the main paper.

S5.5 Singular code for the analysis of a non-RPA-capable bifunctional enzyme (Section S4.5)

```
> ring R = (0,k1,k2,k3,k4,k5,k6,k7,k8,k9,k10,k11,k12,k13,k14,k15,
k16,k17,k18,k19), (x8, x7, x6, x4, x3, x2, x1, x5), (dp(6), dp(2));
```

```
> poly f1 = -k1*x1 + k2*x2 - k3*x1*x5 + k4*x6 + k5*x6 + k13*x4;
> poly f2 = k1*x1 - k2*x2 - k8*x2*x5 + k9*x7 + k10*x7 - k11*x2*x3
                  + k12*x4;
> poly f3 = k5*x6 + k10*x7 - k11*x2*x3 + k12*x4 - k16*x3*x7
                  + k17*x8 + k19*x8;
> poly f4 = k11*x2*x3 - k12*x4 - k14*x4*x5 + k15*x8 - k13*x4 + k19*x8;
> poly f5 = -k3*x1*x5 + k4*x6 + k9*x7 - k8*x2*x5 + k13*x4 - k14*x4*x5
                  + k15*x8 + k18*x8;
> poly f6 = -k6*x6 + k7*x7 - k5*x6 + k3*x1*x5 - k4*x6 + k18*x8;
> poly f7 = k8*x2*x5 - k9*x7 + k6*x6 - k7*x7 - k10*x7 - k16*x7*x3
                  + k17*x8;
> poly f8 = k14*x4*x5 - k15*x8 + k16*x7*x3 - k17*x8 - k18*x8 - k19*x8;
> ideal I = f1, f2, f3, f4, f5, f6, f7, f8;
> ideal GI = groebner(I);
> GI;
```

References

[1] Robyn P Araujo and Lance A Liotta. The topological requirements for robust perfect adaptation in networks of any size. *Nature Communications*, 9(1):1–12, 2018.

[2] Corentin Briat, Ankit Gupta, and Mustafa Khammash. Antithetic integral feedback ensures robust perfect adaptation in noisy biomolecular networks. *Cell systems*, 2(1):15–26, 2016.

[3] Daniele Cappelletti, Ankit Gupta, and Mustafa Khammash. A hidden integral structure endows absolute concentration robust systems with resilience to dynamical concentration

disturbances. *Journal of the Royal Society Interface*, 17(171):20200437, 2020.

[4] David Cox, John Little, and Donal O'Shea. *Ideals, varieties, and algorithms: an introduction to computational algebraic geometry and commutative algebra*. Springer Science, 2013.

[5] Wolfram Decker, Gert-Martin Greuel, Gerhard Pfister, and Hans Schönemann. SINGULAR 4-3-2 — A computer algebra system for polynomial computations, http://www.singular.uni-kl.de, 2022.

[6] Jeanne MO Eloundou-Mbebi, Anika Küken, Nooshin Omranian, Sabrina Kleessen, Jost Neigenfind, Georg Basler, and Zoran Nikoloski. A network property necessary for concentration robustness. *Nature Communications*, 7(1):1–7, 2016.

[7] Martin Feinberg. *Foundations of chemical reaction network theory*. Springer, 2019.

[8] Martin Feinberg and Friedrich JM Horn. Chemical mechanism structure and the coincidence of the stoichiometric and kinetic subspaces. *Archive for Rational Mechanics and Analysis*, 66(1):83–97, 1977.

[9] Bruce A Francis and Walter Murray Wonham. The internal model principle of control theory. *Automatica*, 12(5):457–465, 1976.

[10] Bruce A Francis and William M Wonham. The internal model principle for linear multivariable regulators. *Applied mathematics and optimization*, 2(2):170–194, 1975.

[11] Fritz Horn and Roy Jackson. General mass action kinetics. *Archive for rational mechanics and analysis*, 47(2):81–116, 1972.

[12] Robert L Karp, Mercedes Pérez Millán, Tathagata Dasgupta, Alicia Dickenstein, and Jeremy Gunawardena. Complex-linear invariants of biochemical networks. *Journal of theoretical biology*, 311:130–138, 2012.

[13] Mercedes Pérez Millán, Alicia Dickenstein, Anne Shiu, and Carsten Conradi. Chemical reaction systems with toric steady states. *Bulletin of mathematical biology*, 74(5):1027–1065, 2012.

[14] VA Prabhanjan. *On The Complexity Of Grobner Basis And Border Basis Detection*. PhD thesis, 2013.

[15] David Rolnick and Gwen Spencer. On the robust hardness of gröbner basis computation. *Journal of Pure and Applied Algebra*, 223(5):2080–2100, 2019.

[16] Guy Shinar and Martin Feinberg. Structural sources of robustness in biochemical reaction networks. *Science*, 327(5971):1389–1391, 2010.

[17] Oren Shoval, Uri Alon, and Eduardo Sontag. Symmetry invariance for adapting biological systems. *SIAM Journal on Applied Dynamical Systems*, 10(3):857–886, 2011.

[18] Eduardo D Sontag. Adaptation and regulation with signal detection implies internal model. *Systems & control letters*, 50(2):119–126, 2003.

[19] Bernd Sturmfels and Markus Wiegelmann. Structural Gröbner basis detection. *Applicable Algebra in Engineering, Communication and Computing*, 8(4):257–263, 1997.

[20] Fangzhou Xiao and John C Doyle. Robust perfect adaptation in biomolecular reaction networks. In *2018 IEEE Conference on Decision and Control (CDC)*, pages 4345–4352. IEEE, 2018.
