# Supplementary material for: Universal structures for adaptation in biochemical reaction networks
Source: Nat Commun. 2023 Apr 20;14:2251. doi: 10.1038/s41467-023-38011-9 (PMC10119132; doi:10.1038/s41467-023-38011-9)
Supplement: Supplementary file 2 — Reporting Summary [file 41467_2023_38011_MOESM2_ESM.pdf]

nature portfolio

Corresponding author(s): ROBYN P. ARAUJO, PhD

Last updated by author(s): 6 April, 2023.

Reporting Summary

Nature Portfolio wishes to improve the reproducibility of the work that we publish. This form provides structure for consistency and transparency in reporting. For further information on Nature Portfolio policies, see our Editorial Policies and the Editorial Policy Checklist.

Statistics

For all statistical analyses, confirm that the following items are present in the figure legend, table legend, main text, or Methods section.

n/a	Confirmed	
☒	☐	The exact sample size (*n*) for each experimental group/condition, given as a discrete number and unit of measurement
☒	☐	A statement on whether measurements were taken from distinct samples or whether the same sample was measured repeatedly
☒	☐	The statistical test(s) used AND whether they are one- or two-sided<br>*Only common tests should be described solely by name; describe more complex techniques in the Methods section.*
☒	☐	A description of all covariates tested
☒	☐	A description of any assumptions or corrections, such as tests of normality and adjustment for multiple comparisons
☒	☐	A full description of the statistical parameters including central tendency (e.g. means) or other basic estimates (e.g. regression coefficient) AND variation (e.g. standard deviation) or associated estimates of uncertainty (e.g. confidence intervals)
☒	☐	For null hypothesis testing, the test statistic (e.g. *F*, *t*, *r*) with confidence intervals, effect sizes, degrees of freedom and *P* value noted<br>*Give P values as exact values whenever suitable.*
☒	☐	For Bayesian analysis, information on the choice of priors and Markov chain Monte Carlo settings
☒	☐	For hierarchical and complex designs, identification of the appropriate level for tests and full reporting of outcomes
☒	☐	Estimates of effect sizes (e.g. Cohen's *d*, Pearson's *r*), indicating how they were calculated

Our web collection on statistics for biologists contains articles on many of the points above.

Software and code

Policy information about availability of computer code

Data collection	No data has been collected, generated or referred to in this study.
Data analysis	No data has been analysed in this study. Open-source software Singular (www.Singular.uni-kl.de) was used ONLY for analysis of mathematical equations (polynomials), NOT for analysis of any data. All code provided in Supplementary Information file, along with comprehensive annotations and fully-worked examples. Singular version 4.3.2 has been used to test all equations referenced in this study.

For manuscripts utilizing custom algorithms or software that are central to the research but not yet described in published literature, software must be made available to editors and reviewers. We strongly encourage code deposition in a community repository (e.g. GitHub). See the Nature Portfolio guidelines for submitting code & software for further information.

Data

Policy information about availability of data

All manuscripts must include a data availability statement. This statement should provide the following information, where applicable:

- Accession codes, unique identifiers, or web links for publicly available datasets
- A description of any restrictions on data availability
- For clinical datasets or third party data, please ensure that the statement adheres to our policy

No data has been collected, generated, referred to or analysed in this study.

Human research participants

Policy information about studies involving human research participants and Sex and Gender in Research.

Reporting on sex and gender	No human subjects involved in this study.
Population characteristics	No human subjects involved in this study.
Recruitment	No human subjects involved in this study.
Ethics oversight	No human subjects involved in this study.

Note that full information on the approval of the study protocol must also be provided in the manuscript.

Field-specific reporting

Please select the one below that is the best fit for your research. If you are not sure, read the appropriate sections before making your selection.

[x] Life sciences [ ] Behavioural & social sciences [ ] Ecological, evolutionary & environmental sciences

For a reference copy of the document with all sections, see nature.com/documents/nr-reporting-summary-flat.pdf

Life sciences study design

All studies must disclose on these points even when the disclosure is negative.

Sample size	No data collected or generated in this study. No experiments conducted. No statistical methods required.
Data exclusions	No data collected or generated in this study. No experiments conducted. No statistical methods required.
Replication	No data collected or generated in this study. No experiments conducted. No statistical methods required.
Randomization	No data collected or generated in this study. No experiments conducted. No statistical methods required.
Blinding	No data collected or generated in this study. No experiments conducted. No statistical methods required.

Behavioural & social sciences study design

All studies must disclose on these points even when the disclosure is negative.

Study description	N/A
Research sample	N/A
Sampling strategy	N/A
Data collection	N/A
Timing	N/A
Data exclusions	N/A
Non-participation	N/A
Randomization	N/A

Ecological, evolutionary & environmental sciences study design

All studies must disclose on these points even when the disclosure is negative.

Study description	N/A
Research sample	N/A
Sampling strategy	N/A
Data collection	N/A
Timing and spatial scale	N/A
Data exclusions	N/A
Reproducibility	N/A
Randomization	N/A
Blinding	N/A

Did the study involve field work? ☐ Yes ☒ No

Field work, collection and transport

Field conditions	N/A
Location	N/A
Access & import/export	N/A
Disturbance	N/A

Reporting for specific materials, systems and methods

We require information from authors about some types of materials, experimental systems and methods used in many studies. Here, indicate whether each material, system or method listed is relevant to your study. If you are not sure if a list item applies to your research, read the appropriate section before selecting a response.

Materials & experimental systems

n/a	Involved in the study	
☒	☐	Antibodies
☒	☐	Eukaryotic cell lines
☒	☐	Palaeontology and archaeology
☒	☐	Animals and other organisms
☒	☐	Clinical data
☒	☐	Dual use research of concern

Methods

n/a	Involved in the study	
☒	☐	ChIP-seq
☒	☐	Flow cytometry
☒	☐	MRI-based neuroimaging

Antibodies

Antibodies used	No antibodies used in this study.
Validation	No antibodies used in this study.

Eukaryotic cell lines

Policy information about cell lines and Sex and Gender in Research

Cell line source(s)	No cell lines used in this study.
Authentication	No cell lines used in this study.
Mycoplasma contamination	No cell lines used in this study.
Commonly misidentified lines (See ICLAC register)	No cell lines used in this study.

Palaeontology and Archaeology

Specimen provenance	N/A
Specimen deposition	N/A
Dating methods	N/A

☐ Tick this box to confirm that the raw and calibrated dates are available in the paper or in Supplementary Information.

Ethics oversight	N/A

Note that full information on the approval of the study protocol must also be provided in the manuscript.

Animals and other research organisms

Policy information about studies involving animals; ARRIVE guidelines recommended for reporting animal research, and Sex and Gender in Research

Laboratory animals	N/A The study did not involve animals.
Wild animals	N/A The study did not involve animals.
Reporting on sex	N/A The study did not involve animals.
Field-collected samples	N/A The study did not involve animals.
Ethics oversight	N/A The study did not involve animals.

Note that full information on the approval of the study protocol must also be provided in the manuscript.

Clinical data

Policy information about clinical studies

All manuscripts should comply with the ICMJEguidelines for publication of clinical research and a completedCONSORT checklist must be included with all submissions.

Clinical trial registration	N/A. No clinical data involved in this study.
Study protocol	N/A. No clinical data involved in this study.
Data collection	N/A. No clinical data involved in this study.
Outcomes	N/A. No clinical data involved in this study.

Dual use research of concern

Policy information about dual use research of concern

Hazards

Could the accidental, deliberate or reckless misuse of agents or technologies generated in the work, or the application of information presented in the manuscript, pose a threat to:

No	Yes	
☒	☐	Public health
☒	☐	National security
☒	☐	Crops and/or livestock
☒	☐	Ecosystems
☒	☐	Any other significant area

Experiments of concern

Does the work involve any of these experiments of concern:

No	Yes	
☒	☐	Demonstrate how to render a vaccine ineffective
☒	☐	Confer resistance to therapeutically useful antibiotics or antiviral agents
☒	☐	Enhance the virulence of a pathogen or render a nonpathogen virulent
☒	☐	Increase transmissibility of a pathogen
☒	☐	Alter the host range of a pathogen
☒	☐	Enable evasion of diagnostic/detection modalities
☒	☐	Enable the weaponization of a biological agent or toxin
☒	☐	Any other potentially harmful combination of experiments and agents

ChIP-seq

Data deposition

☐ Confirm that both raw and final processed data have been deposited in a public database such as GEO.

☐ Confirm that you have deposited or provided access to graph files (e.g. BED files) for the called peaks.

Data access links<br>*May remain private before publication.*	N/A. No ChIP-seq involved in this study.
Files in database submission	N/A. No ChIP-seq involved in this study.
Genome browser session<br>(e.g. UCSC)	N/A. No ChIP-seq involved in this study.

Methodology

Replicates	N/A. No ChIP-seq involved in this study.
Sequencing depth	N/A. No ChIP-seq involved in this study.
Antibodies	N/A. No ChIP-seq involved in this study.
Peak calling parameters	N/A. No ChIP-seq involved in this study.
Data quality	N/A. No ChIP-seq involved in this study.
Software	N/A. No ChIP-seq involved in this study.

Flow Cytometry

Plots

Confirm that:

☐ The axis labels state the marker and fluorochrome used (e.g. CD4-FITC).

☐ The axis scales are clearly visible. Include numbers along axes only for bottom left plot of group (a 'group' is an analysis of identical markers).

☐ All plots are contour plots with outliers or pseudocolor plots.

☐ A numerical value for number of cells or percentage (with statistics) is provided.

Methodology

Sample preparation	N/A. No flow cytometry involved in this study.
Instrument	N/A. No flow cytometry involved in this study.
Software	N/A. No flow cytometry involved in this study.
Cell population abundance	N/A. No flow cytometry involved in this study.
Gating strategy	N/A. No flow cytometry involved in this study.

☐ Tick this box to confirm that a figure exemplifying the gating strategy is provided in the Supplementary Information.

Magnetic resonance imaging

Experimental design

Design type	N/A. No MRI used in this study.
Design specifications	N/A. No MRI used in this study.
Behavioral performance measures	N/A. No MRI used in this study.

Acquisition

Imaging type(s)	N/A. No MRI used in this study.
Field strength	N/A. No MRI used in this study.
Sequence & imaging parameters	N/A. No MRI used in this study.
Area of acquisition	N/A. No MRI used in this study.

Diffusion MRI ☐ Used ☒ Not used

Preprocessing

Preprocessing software	N/A. No MRI used in this study.
Normalization	N/A. No MRI used in this study.
Normalization template	N/A. No MRI used in this study.
Noise and artifact removal	N/A. No MRI used in this study.
Volume censoring	N/A. No MRI used in this study.

Statistical modeling & inference

Model type and settings	N/A. No MRI used in this study.
Effect(s) tested	N/A. No MRI used in this study.

Specify type of analysis: ☐ Whole brain ☐ ROI-based ☐ Both

Statistic type for inference (See Eklund et al. 2016)	N/A. No MRI used in this study.
Correction	N/A. No MRI used in this study.

Models & analysis

n/a	Involved in the study
☒	☐ Functional and/or effective connectivity
☒	☐ Graph analysis
☒	☐ Multivariate modeling or predictive analysis

Functional and/or effective connectivity	N/A. No MRI used in this study.
Graph analysis	N/A. No MRI used in this study.
Multivariate modeling and predictive analysis	N/A. No MRI used in this study.
